# Supplementary material for: CD157+ vascular endothelial stem cells represent a conserved subpopulation with an angiogenic gene expression profile
Source: Stem Cell Reports. 2026 Jun 4;21(7):102931. doi: 10.1016/j.stemcr.2026.102931 (PMC13385430; doi:10.1016/j.stemcr.2026.102931)
Supplement: Document S1. Figures S1–S7 and Tables S1 and S2 [file mmc1.pdf]

**Stem Cell Reports, Volume *21***

## Supplemental Information

# CD157$^+$ vascular endothelial stem cells represent a conserved sub-population with an angiogenic gene expression profile

**Tomohiro Iba, Taku Wakabayashi, Rie Ito, Ai Sugawara, Satoshi Fujimura, Mika Sawane, Kazuaki Yoshioka, Aya Matsui, Jun-ichi Morishige, Naoto Nagata, Yukinobu Ito, Masafumi Horie, Daichi Maeda, Rica Tanaka, Hitoshi Ando, Nobuyuki Takakura, and Hisamichi Naito**

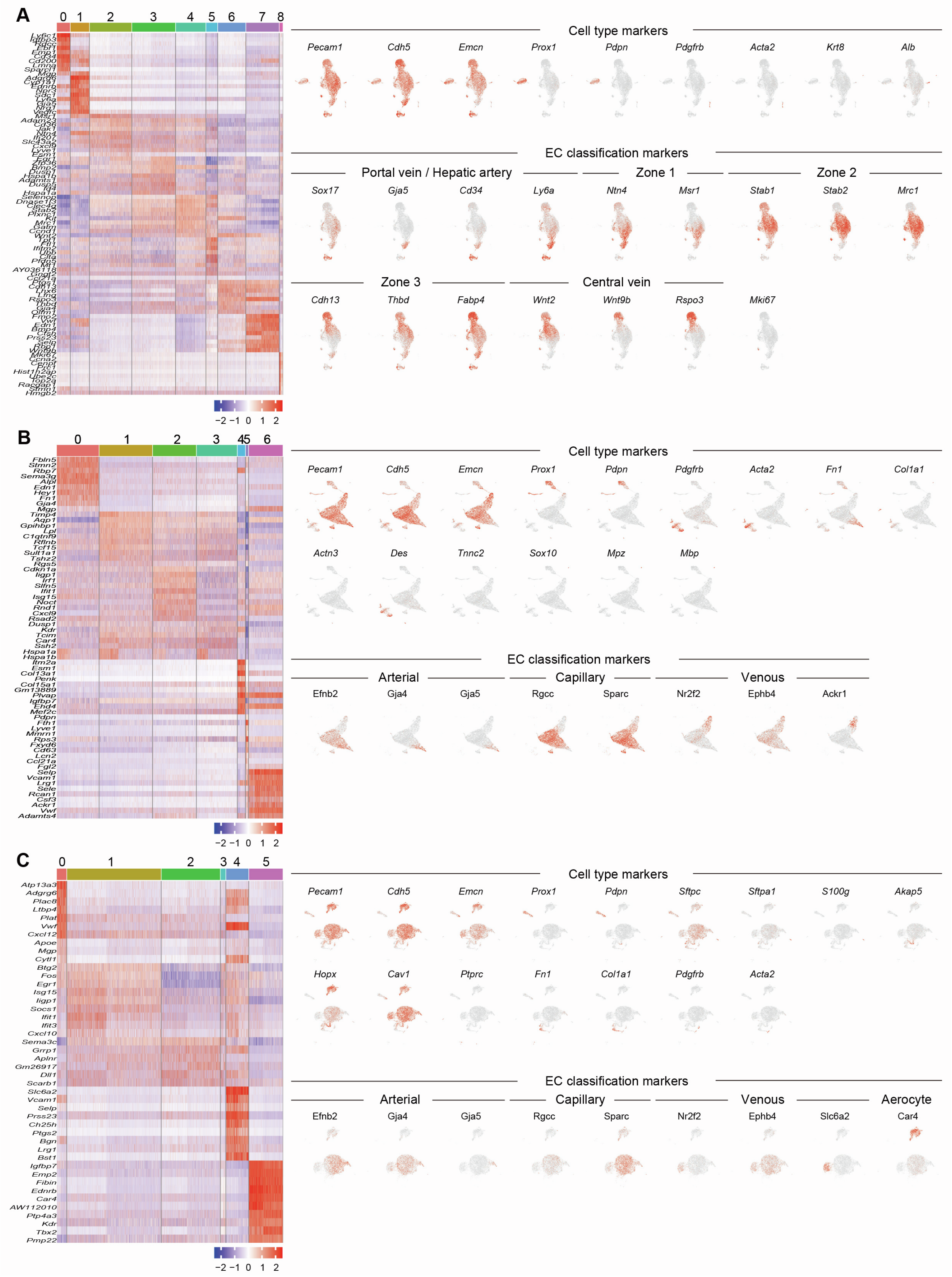


**Figure S1. Characterization of EC subclusters in each mouse tissue**

Heatmaps (left panels) showing the top 10 differentially expressed genes in each cluster of CD31$^{+}$CD45$^{-}$ sorted cells from mouse liver (**A**), hindlimb muscle (**B**), and lung (**C**), as shown in **Figure 1**. Feature plots of selected cell type annotation markers and endothelial cell (EC) subpopulation markers are shown on the right.

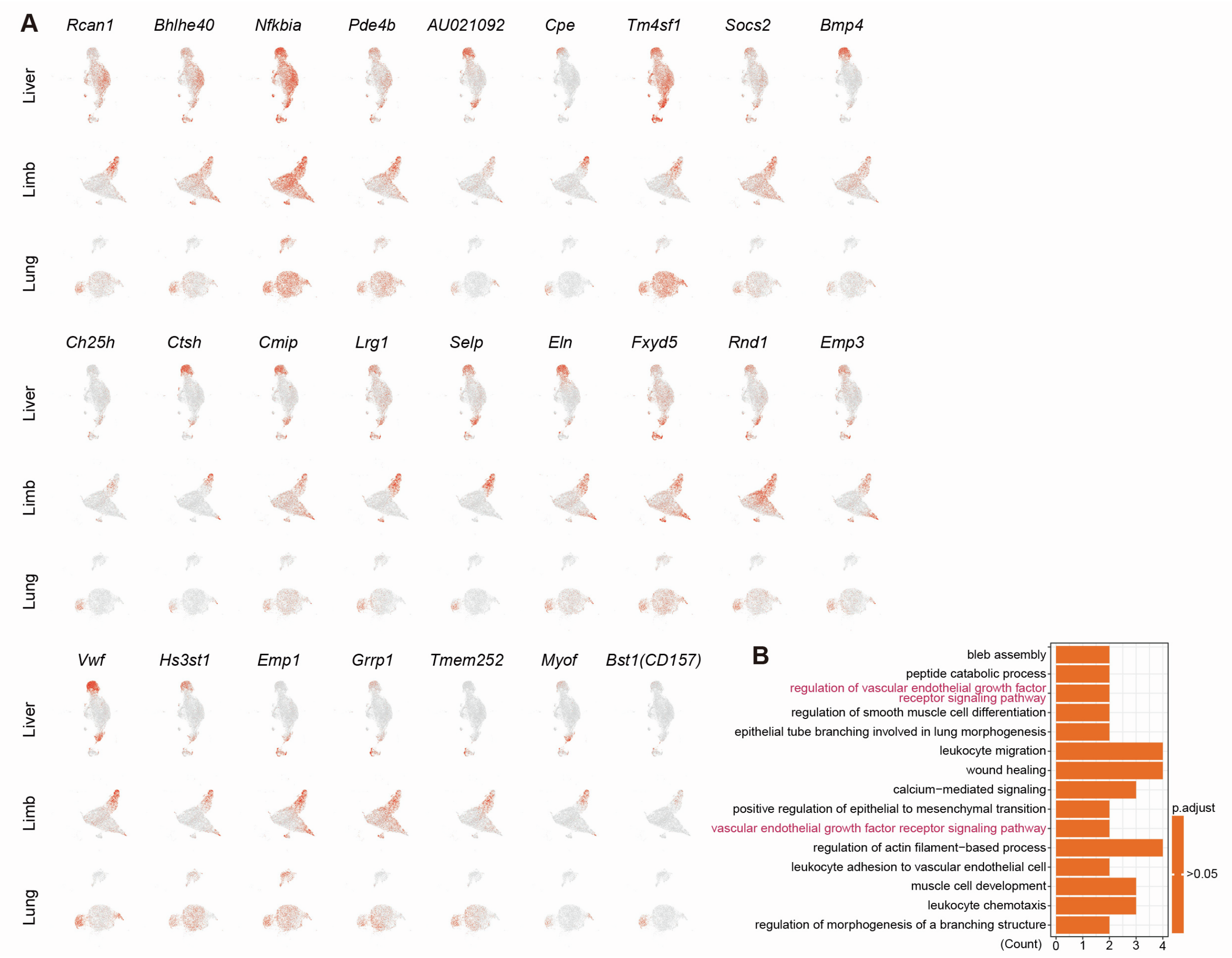


**Figure S2. Analysis of genes shared by *CD157*-positive ECs across mouse tissues**

**A.** Feature plots showing the expression of genes commonly expressed in *CD157*-positive ECs across mouse tissues (identified in **Figure 2C**) in the mouse liver, hindlimb muscle, and lung.

**B.** GO analysis of genes shown in **Figure S2A**.

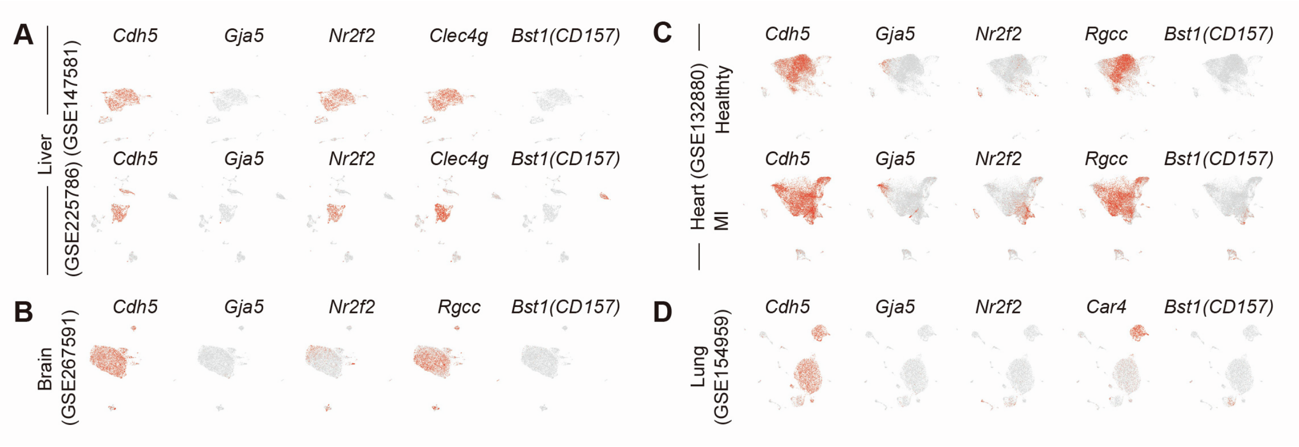


**Figure S3. Reanalysis of public mouse scRNA-seq datasets**

Feature plots showing the expression of the pan-endothelial marker *Cdh5*; arterial marker *Gja5*; venous marker *Nr2f2*; organ-specific EC markers—*Clec4g* for liver sinusoidal ECs, *Rgcc* for heart capillary ECs, and *Car4* for lung aCaps—as well as *Bst1* (*CD157*), in the liver (**A**), brain (**B**) heart under healthy and myocardial infarction (MI) conditions (**C**), and lung (**D**). This analysis was based on publicly accessible mouse scRNA-seq datasets obtained from the following repositories: liver (GSE147581, GSE225786), brain (GSE267591), heart (GSE132880), and lung (GSE154959).

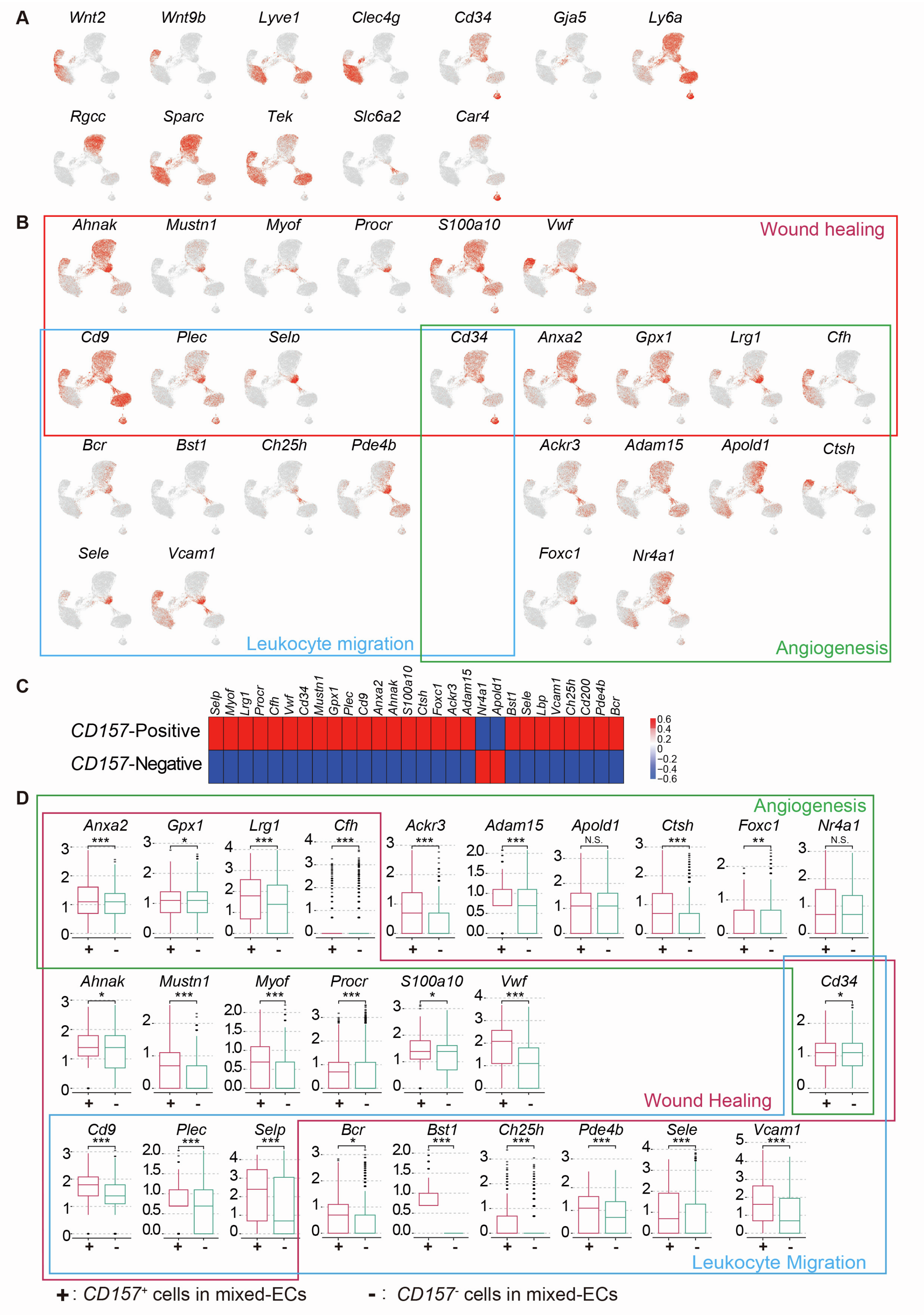
A
Wnt2
Wnt9b
Lyve1
Clec4g
Cd34
Gja5
Ly6a
Rgcc
Sparc
Tek
Slc6a2
Car4
B
Wound healing
Ahnak
Mustn1
Myof
Procr
S100a10
Vwf
Cd9
Plec
Selp
Cd34
Anxa2
Gpx1
Lrg1
Cfh
Bcr
Bst1
Ch25h
Pde4b
Ackr3
Adam15
Apold1
Ctsh
Sele
Vcam1
Foxc1
Nr4a1
Leukocyte migration
Angiogenesis
C
CD157-Positive
CD157-Negative
D
Angiogenesis
Wound Healing
Leukocyte Migration
+: CD157+ cells in mixed-ECs
-: CD157- cells in mixed-ECs

**Figure S4. Characterization of *CD157*-positive and *CD157*-negative ECs using integrated EC datasets**

**A.** Feature plots showing the expression of central vein markers (*Wnt2* and *Wnt9b*), sinusoidal EC markers (*Lyve1* and *Clec4g*), portal vein EC marker *Cd34*, arterial EC marker *Gja5*, capillary markers (*Rgcc* and *Sparc*), pulmonary general capillary (gCap) marker (*Tek*), pulmonary venous marker (*Slc6a2*), and aerocyte marker (*Car4*) are projected onto the Uniform Manifold Approximation and Projection (UMAP) plots of ECs integrated from three mouse tissues.

**B.** Feature plots showing the expression of genes associated with the *CD157*-positive cell-specific pathway identified in **Figure 3H**. Genes related to wound healing are outlined in red, those related to leukocyte migration in blue, and those related to angiogenesis in green.

**C**. Heatmap of genes shown in **Figure 3G**, comparing *CD157*-positive and *CD157*-negative ECs within the mixed-EC cluster.

**D**. Box plots showing expression of genes detected by pathway analysis in **Figure 3H**, based on the gene set defined in **Figure 3G**, comparing *CD157*-positive and *CD157*-negative ECs within the mixed-EC cluster. Genes related to wound healing are outlined in red, those related to leukocyte migration in blue, and those related to angiogenesis in green. *** $p < 0.0005$, ** $p < 0.005$, *$p < 0.05$.

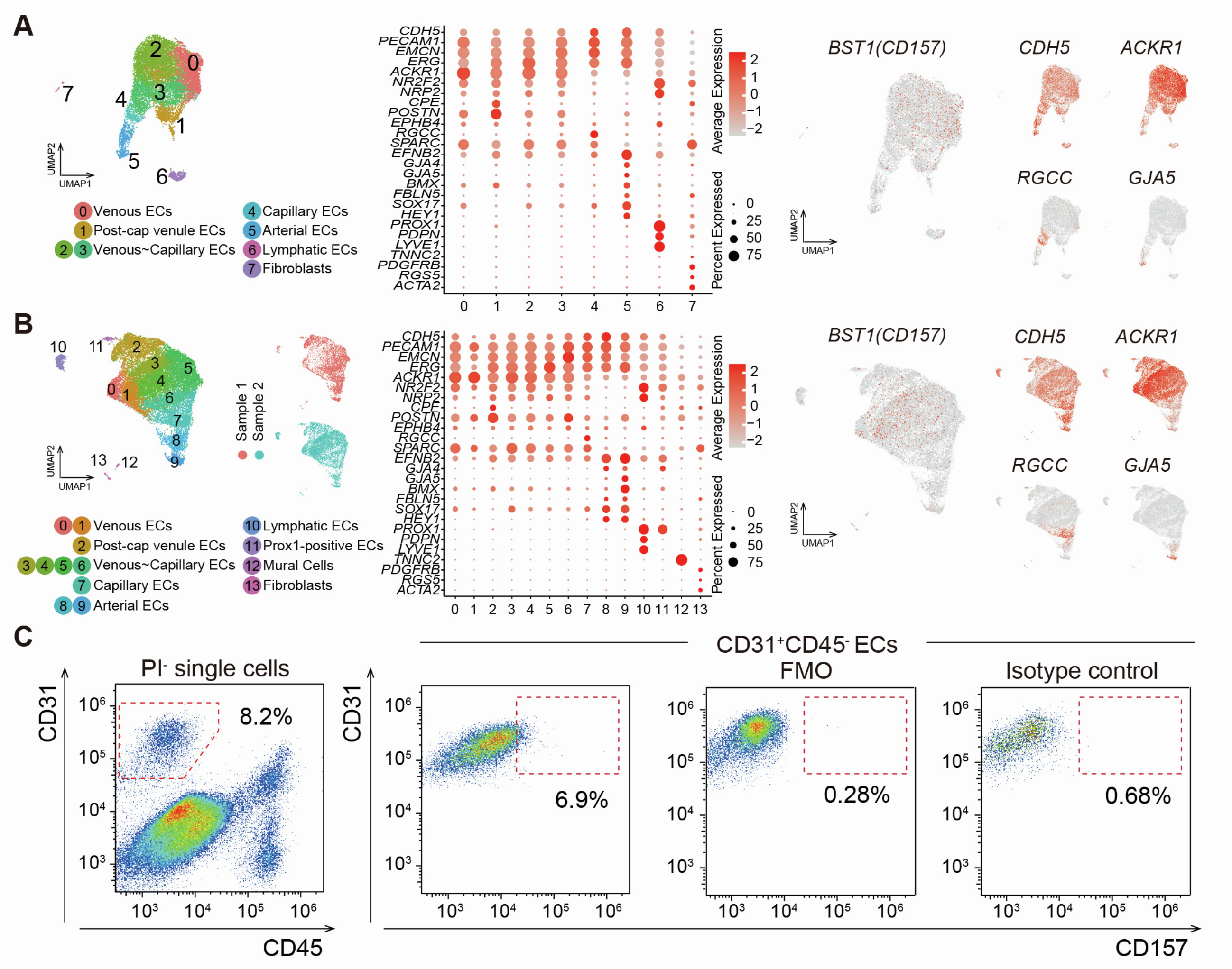


**Figure S5. Bioinformatic characterization of human muscle EC single-cell data and FACS profiling of CD157$^{+}$ ECs**

**A.** scRNA-seq analysis of an independent dataset of human muscle CD31$^{+}$CD45$^{-}$ cells, separate from that used in **Figure 5**. (Left) UMAP plots and clustering of 12,915 cells. (Middle) Bubble plots showing expression of canonical marker genes across clusters. (Right) Feature plots of *BST1* (*CD157*), *CDH5*, *ACKR1*, *RGCC*, and *GJA5*.

**B.** Integrated scRNA-seq analysis of a human muscle CD31$^{+}$CD45$^{-}$ cell dataset shown in **Figure 5** and the independent dataset shown in **Figure S5A**. (Left) UMAP plots and clustering of the integrated 17,873 cells. (Middle) Bubble plots showing expression of canonical marker genes across clusters. (Right) Feature plots of *BST1* (*CD157*), *CDH5*, *ACKR1*, *RGCC*, and *GJA5*.

**C.** Representative flow cytometry dot plots of cells isolated from human lower limb muscle, showing CD157 antibody staining with fluorescence minus one (FMO) and isotype controls. The red gate indicates CD157$^{+}$ cells used for cell culture assays.

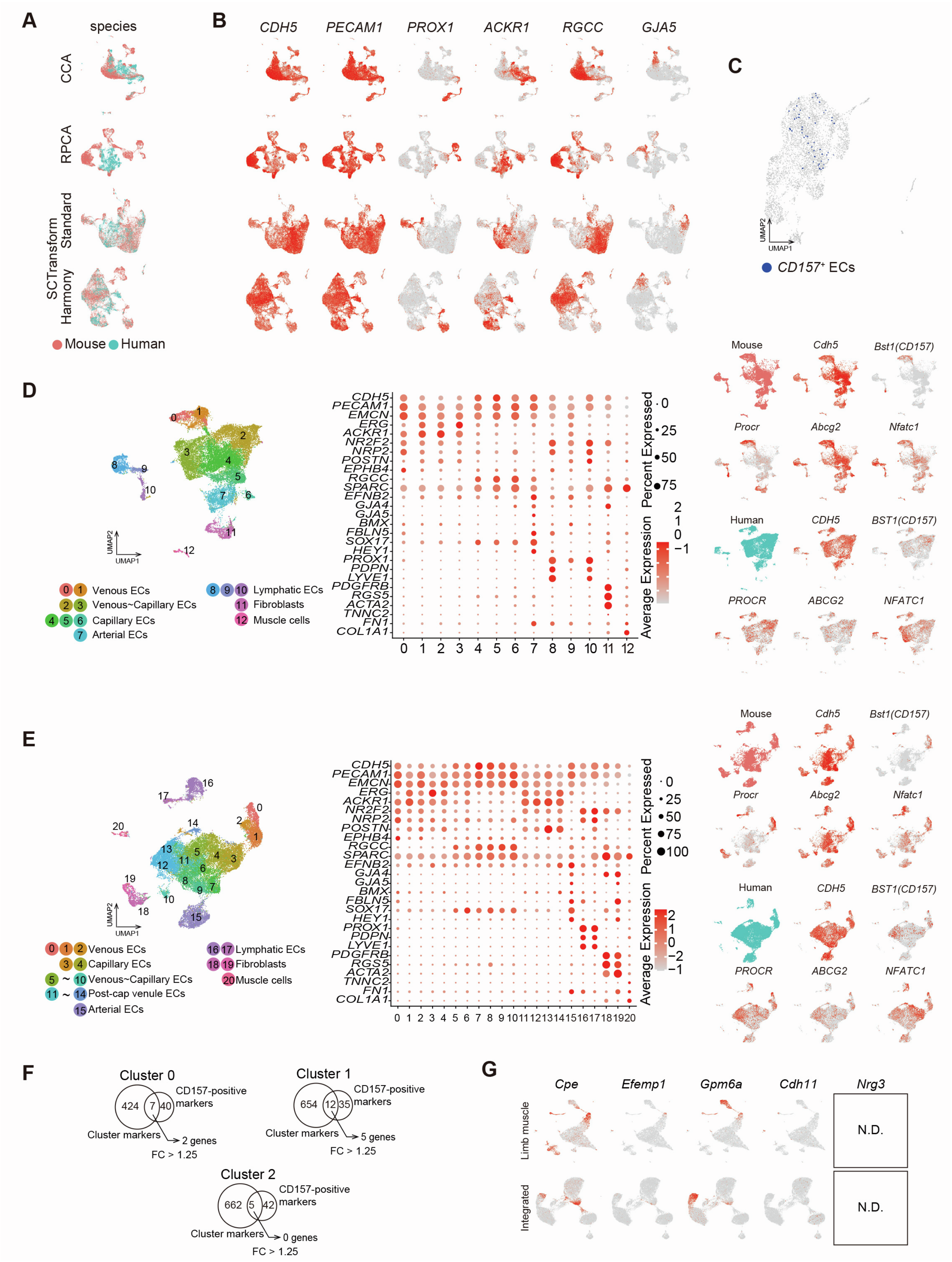
A
species
CCA
RPCA
SCTransform Standard
Harmony
Mouse
Human
B
CDH5
PECAM1
PROX1
ACKR1
RGCC
GJA5
C
UMAP2
UMAP1
CD157⁺ ECs
Mouse
Cdh5
Bst1(CD157)
Procr
Abcg2
Nfatc1
Human
CDH5
BST1(CD157)
PROCR
ABCG2
NFATC1
D
UMAP2
UMAP1
0 1 Venous ECs
2 3 Venous~Capillary ECs
4 5 6 Capillary ECs
7 Arterial ECs
8 9 10 Lymphatic ECs
11 Fibroblasts
12 Muscle cells
CDH5
PECAM1
EMCN
ERG
ACKR1
NR2F2
NRP2
POSTN
EPHB4
RGCC
SPARC
EFNB2
GJA4
GJA5
BMX
FBLN5
SOX17
HEY1
PROX1
PDPN
LYVE1
PDGFRB
RGS5
ACTA2
TNNC2
FN1
COL1A1
0 1 2 3 4 5 6 7 8 9 10 11 12
Percent Expressed
0
25
50
75
Average Expression
2
1
0
−1
E
UMAP2
UMAP1
0 1 2 Venous ECs
3 4 Capillary ECs
5 ~ 10 Venous~Capillary ECs
11 ~ 14 Post-cap venule ECs
15 Arterial ECs
16 17 Lymphatic ECs
18 19 Fibroblasts
20 Muscle cells
0 1 2 3 4 5 6 7 8 9 10 11 12 13 14 15 16 17 18 19 20
Percent Expressed
0
25
50
75
100
Average Expression
2
1
0
−2
−1
F
Cluster 0
424
7
40
CD157-positive markers
Cluster markers
2 genes
FC > 1.25
Cluster 1
654
12
35
CD157-positive markers
Cluster markers
5 genes
FC > 1.25
Cluster 2
662
5
42
CD157-positive markers
Cluster markers
0 genes
FC > 1.25
G
Cpe
Efemp1
Gpm6a
Cdh11
Nrg3
Limb muscle
Integrated
N.D.
N.D.

**Figure S6. Comparison of human–mouse integration methods and gene expression similarity**

**A.** UMAP plots of human and mouse datasets integrated using multiple methods. Dot colors indicate species origin.

**B.** Feature plots of the indicated genes used to define EC subsets, projected onto the UMAP plots shown in **Figure S6A.**

**C.** Feature plots of *CD157*-positive cells extracted from clusters 0, 1, and 2 of the integrated dataset and re-projected onto the human UMAP shown in **Figure 5A**.

**D.** Integrated scRNA-seq analysis combining a human muscle $CD31^{+}CD45^{-}$ cell dataset used in **Figure S5A** (12,915 cells; distinct from the dataset used in **Figure 6**) with the mouse hindlimb $CD31^{+}CD45^{-}$ cell dataset used in **Figure 6** (12,681 cells). (Left) UMAP plots and clustering of the integrated data. (Middle) Bubble plots showing expression of canonical marker genes across clusters.(Right) Feature plots of indicated genes projected onto species-split UMAPs of the integrated dataset.

**E.** Integrated scRNA-seq analysis combining the two human datasets used in **Figure S5B** (total 17,873 cells; from **Figures 5** and **S5A**) with the mouse hindlimb $CD31^{+}CD45^{-}$ cell dataset used in **Figure 6** (12,681 cells). (Left) UMAP plots and clustering of the integrated data.(Middle) Bubble plots showing expression of canonical marker genes across clusters. (Right) Feature plots of indicated genes projected onto species-split UMAPs of the integrated dataset.

**F.** Venn diagrams showing the overlap of upregulated genes between each cluster and *CD157*-positive ECs.

**G.** Feature plots of Cluster 0 signature genes in the mouse limb muscle dataset (top) and in the mouse integrated dataset (bottom).

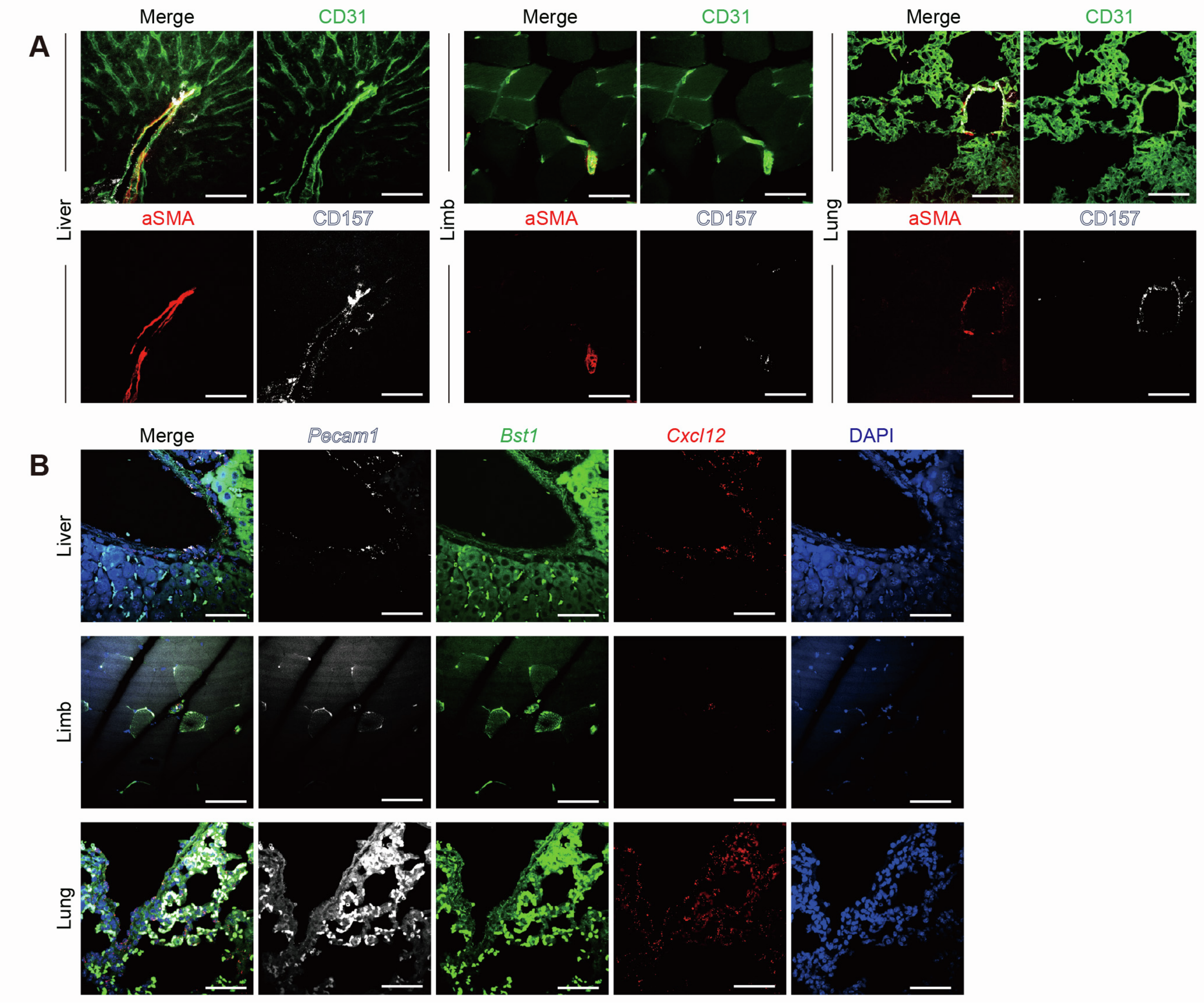


**Figure S7. Perivascular expression of CXCL12 by in situ hybridization**

**A.** Immunofluorescence images of mouse liver, hindlimb muscle, and lung tissues stained for CD31 (green), αSMA (red), and CD157 (white), showing smooth muscle cells surrounding the CD31$^{+}$ endothelium, with CD157 expression observed in a subset of ECs. Scale bars: 50 μm.

**B.** *In situ* hybridization for *Pecam1* together with *Bst1* and *Cxcl12* in mouse liver, hindlimb muscle, and lung tissues. *Cxcl12* signals are observed in regions corresponding to the αSMA-positive areas shown in **Figure S7A**. Signals for *Pecam1* (white), *Bst1* (green), *Cxcl12* (red), and DAPI (blue) are shown. Scale bars: 50 μm.

| Cluster 0 | Cluster 1 | Cluster 2 |
|---|---|---|
| CPE | SELE | DOCK11 |
| EFEMP1 | CSF3 | PLCXD3 |
| MGP | IL6 | HLA-DQA1 |
| GPM6A | VCAM1 | NET1 |
| CDH11 | SLC4A7 | LHX6 |
| CRTAC1 | TMEM70 | KRT7 |
| MMRN1 | ADAMTS3 | PDE7B |
| TMOD1 | CYP1B1 | C7 |
| PTGIS | CFAP161 | |
| PLA2G4A | RGS16 | |
| NRG3 | PVT1 | |
| CARMIL1 | TRIB1 | |
| MEIS2 | STC1 | |
| TIMP1 | ENKUR | |
| RAB11FIP1 | C7 | |
| C7 | SLC16A7 | |
| SLCO2A1 | SELP | |
| PTGS2 | SLC6A16 | |
| CYP1B1 | RHOU | |
| ACKR3 | LRG1 | |
| ST8SIA6 | LUCAT1 | |
| SRPX | RHBDL2 | |
| HS3ST2 | KDM6B | |
| HMGN3 | HAPLN3 | |
| ITGA10 | | |
| NR2F2-AS1 | | |
| CTSC | | |
| PLCXD3 | | |
| PON2 | | |
| LDLRAD3 | | |
| IL13RA1 | | |
| BMX | | |
| NPAS2 | | |
| RYR3 | | |
| COL8A1 | | |
| CAMK1D | | |
| HRCT1 | | |
| CSGALNACT1 | | |
| PDLIM3 | | |
| C11orf96 | | |
| SGMS2 | | |

**Table S1. Differentially expressed genes in human EC clusters 0, 1, and 2**

List of differentially expressed genes (DEGs) in human EC clusters 0, 1, and 2 from the integrated dataset. DEGs were selected based on the criteria of fold change (FC) > 1.25 and expression detected in less than 50% of cells in the comparison group (pct.2 < 0.5).

| | CD157+ ECs | CD157- ECs | Liver ECs_1 | Liver ECs_2 | Liver ECs_3 | Liver ECs_4 | Central vein | Limb capillary ECs | Lung capillary ECs | aCaps | Lymphatic ECs | Macrophages | alveolar macrophages | Neutrophils | T cells | NKTs | B cells | Plasma cells | Pericytes/SMCs | Hepatocytes | Type1 epithelium | Type2 epithelium | Cholangiocytes | Ciliated cells | Fibroblasts |
|---|---|---|---|---|---|---|---|---|---|---|---|---|---|---|---|---|---|---|---|---|---|---|---|---|---|
| CD157+ ECs | 0.02293434 | 0.01670262 | 0 | 0 | 0 | 0 | 0.0011429 | 0.002642633 | 0.002624418 | 0 | 0.002595711 | 0 | 0 | 0.01812082 | 0.00215042 | 0 | 0 | 0 | 0 | 0 | 0 | 0 | 0 | 0 | 0 |
| CD157- ECs | 0.03936366 | 0.03410016 | 0 | 0 | 0 | 0 | 0.00130212 | 0.003010162 | 0.00298942 | 0 | 0.002956733 | 0 | 0 | 0.02541389 | 0.00270447 | 0 | 0 | 0 | 0 | 0 | 0 | 0 | 0 | 0 | 0 |
| Liver ECs_1 | 0.00899635 | 0.01024146 | 0 | 0 | 0 | 0 | 0 | 0 | 0 | 0 | 0 | 0 | 0 | 0 | 0.01121818 | 0 | 0 | 0 | 0 | 0 | 0 | 0 | 0 | 0 | 0 |
| Liver ECs_2 | 0.00355573 | 0.00404838 | 0 | 0 | 0 | 0 | 0 | 0 | 0 | 0 | 0 | 0 | 0 | 0 | 0.0039095 | 0 | 0 | 0 | 0 | 0 | 0 | 0 | 0 | 0 | 0 |
| Liver ECs_3 | 0 | 0 | 0 | 0 | 0 | 0 | 0 | 0 | 0 | 0 | 0 | 0 | 0 | 0 | 0 | 0 | 0 | 0 | 0 | 0 | 0 | 0 | 0 | 0 | 0 |
| Liver ECs_4 | 0.01992636 | 0.02266427 | 0 | 0 | 0 | 0 | 0 | 0 | 0 | 0 | 0 | 0 | 0 | 0 | 0.00433206 | 0 | 0 | 0 | 0 | 0 | 0 | 0 | 0 | 0 | 0 |
| Central vein | 0.00322002 | 0.00366634 | 0 | 0 | 0 | 0 | 0 | 0 | 0 | 0 | 0 | 0 | 0 | 0 | 0.00715847 | 0 | 0 | 0 | 0 | 0 | 0 | 0 | 0 | 0 | 0 |
| Limb capillary ECs | 0.10122095 | 0.0494083 | 0 | 0 | 0 | 0 | 0.00895295 | 0.02049096 | 0.020352226 | 0 | 0.020133521 | 0 | 0 | 0.0422096 | 0 | 0 | 0 | 0 | 0 | 0 | 0 | 0 | 0 | 0 | 0 |
| Lung capillary ECs | 0.04530587 | 0.02024101 | 0 | 0 | 0 | 0 | 0.00397006 | 0.009145687 | 0.009083054 | 0 | 0.008984337 | 0 | 0 | 0.01509177 | 0 | 0 | 0 | 0 | 0 | 0 | 0 | 0 | 0 | 0 | 0 |
| aCaps | 0 | 0 | 0 | 0 | 0 | 0 | 0 | 0 | 0 | 0 | 0 | 0 | 0 | 0 | 0.0024613 | 0 | 0 | 0 | 0 | 0 | 0 | 0 | 0 | 0 | 0 |
| Lymphatic ECs | 0 | 0 | 0 | 0 | 0 | 0 | 0 | 0 | 0 | 0 | 0 | 0 | 0 | 0 | 0 | 0 | 0 | 0 | 0 | 0 | 0 | 0 | 0 | 0 | 0 |
| Macrophages | 0 | 0 | 0 | 0 | 0 | 0 | 0 | 0 | 0 | 0 | 0 | 0 | 0 | 0 | 0.00430749 | 0 | 0 | 0 | 0 | 0 | 0 | 0 | 0 | 0 | 0 |
| alveolar macrophages | 0 | 0 | 0 | 0 | 0 | 0 | 0 | 0 | 0 | 0 | 0 | 0 | 0 | 0 | 0 | 0 | 0 | 0 | 0 | 0 | 0 | 0 | 0 | 0 | 0 |
| Neutrophils | 0 | 0 | 0 | 0 | 0 | 0 | 0 | 0 | 0 | 0 | 0 | 0 | 0 | 0 | 0 | 0 | 0 | 0 | 0 | 0 | 0 | 0 | 0 | 0 | 0 |
| T cells | 0 | 0 | 0 | 0 | 0 | 0 | 0 | 0 | 0 | 0 | 0 | 0 | 0 | 0 | 0 | 0 | 0 | 0 | 0 | 0 | 0 | 0 | 0 | 0 | 0 |
| NKTs | 0 | 0 | 0 | 0 | 0 | 0 | 0 | 0 | 0 | 0 | 0 | 0 | 0 | 0 | 0 | 0 | 0 | 0 | 0 | 0 | 0 | 0 | 0 | 0 | 0 |
| B cells | 0 | 0 | 0 | 0 | 0 | 0 | 0 | 0 | 0 | 0 | 0 | 0 | 0 | 0 | 0 | 0 | 0 | 0 | 0 | 0 | 0 | 0 | 0 | 0 | 0 |
| Plasma cells | 0 | 0 | 0 | 0 | 0 | 0 | 0 | 0 | 0 | 0 | 0 | 0 | 0 | 0 | 0.00191311 | 0 | 0 | 0 | 0 | 0 | 0 | 0 | 0 | 0 | 0 |
| Pericytes/SMCs | 0.08351968 | 0.04150379 | 0 | 0 | 0 | 0 | 0.00709958 | 0.016288335 | 0.016177584 | 0 | 0.016003007 | 0 | 0 | 0.05100127 | 0 | 0 | 0 | 0 | 0 | 0 | 0 | 0 | 0 | 0 | 0 |
| Hepatocytes | 0 | 0 | 0 | 0 | 0 | 0 | 0 | 0 | 0 | 0 | 0 | 0 | 0 | 0 | 0 | 0 | 0 | 0 | 0 | 0 | 0 | 0 | 0 | 0 | 0 |
| Type1 epithelium | 0 | 0 | 0 | 0 | 0 | 0 | 0 | 0 | 0 | 0 | 0 | 0 | 0 | 0 | 0.00445953 | 0 | 0 | 0 | 0 | 0 | 0 | 0 | 0 | 0 | 0 |
| Type2 epithelium | 0 | 0 | 0 | 0 | 0 | 0 | 0 | 0 | 0 | 0 | 0 | 0 | 0 | 0 | 0.00201727 | 0 | 0 | 0 | 0 | 0 | 0 | 0 | 0 | 0 | 0 |
| Cholangiocytes | 0 | 0 | 0 | 0 | 0 | 0 | 0 | 0 | 0 | 0 | 0 | 0 | 0 | 0 | 0 | 0 | 0 | 0 | 0 | 0 | 0 | 0 | 0 | 0 | 0 |
| Ciliated cells | 0 | 0 | 0 | 0 | 0 | 0 | 0 | 0 | 0 | 0 | 0 | 0 | 0 | 0 | 0 | 0 | 0 | 0 | 0 | 0 | 0 | 0 | 0 | 0 | 0 |
| Fibroblasts | 0.02113242 | 0.01257721 | 0 | 0 | 0 | 0 | 0.00140049 | 0.003237147 | 0.003214846 | 0 | 0.003179702 | 0 | 0 | 0.02793045 | 0 | 0 | 0 | 0 | 0 | 0 | 0 | 0 | 0 | 0 | 0 |

**Table S2. Communication probability scores of CXCL signaling between clusters**

A table of communication probability scores. Scores between *CD157*-positive ECs and pericytes/SMCs, as well as those between *CD157*-negative ECs and pericytes/SMCs, are highlighted.
