# Supplementary material for: CD157+ vascular endothelial stem cells represent a conserved subpopulation with an angiogenic gene expression profile
Source: Stem Cell Reports. 2026 Jun 4;21(7):102931. doi: 10.1016/j.stemcr.2026.102931 (PMC13385430; doi:10.1016/j.stemcr.2026.102931)
Supplement: Document S2. Article plus supplemental information [file mmc2.pdf]

STEM CELL REPORTS
Article
OPEN ACCESS

CD157$^+$ vascular endothelial stem cells represent a conserved subpopulation with an angiogenic gene expression profile

Tomohiro Iba,[1,2] Taku Wakabayashi,[1] Rie Ito,[3] Ai Sugawara,[3,4] Satoshi Fujimura,[3,4] Mika Sawane,[5] Kazuaki Yoshioka,[1] Aya Matsui,[1] Jun-ichi Morishige,[2] Naoto Nagata,[2] Yukinobu Ito,[6] Masafumi Horie,[7] Daichi Maeda,[6,7,8] Rica Tanaka,[3,4,9] Hitoshi Ando,[2] Nobuyuki Takakura,[10,11,*] and Hisamichi Naito[1,12,*]
[1]Department of Vascular Physiology, Graduate School of Medical Science, Kanazawa University, 13-1, Takara-machi, Kanazawa, Ishikawa 920-8640, Japan
[2]Department of Cellular and Molecular Function Analysis, Graduate School of Medical Science, Kanazawa University, 13-1, Takara-machi, Kanazawa, Ishikawa 920-8640, Japan
[3]Division of Regenerative Therapy, Juntendo University Graduate School of Medicine, 2-1-1, Hongo, Bunkyo-ku, Tokyo 113-8421, Japan
[4]Intractable Disease Research Center, Juntendo University Graduate School of Medicine, 2-1-1, Hongo, Bunkyo-ku, Tokyo 113-8421, Japan
[5]Shiseido Global Innovation Center, Yokohama 220-8559, Japan
[6]Department of Molecular and Cellular Pathology, Kanazawa University Graduate School of Medical Sciences, 13-1 Takaramachi, Kanazawa, Ishikawa 920-8640, Japan
[7]Division of Molecular and Genomic Pathology, Department of Pathology, Kobe University Graduate School of Medicine, 7-5-1 Kusunoki-cho, Chuo-ku, Kobe 650-0017, Japan
[8]Garvan Institute of Medical Research, 384 Victoria Street, Darlinghurst, NSW 2010, Australia
[9]Department of Plastic and Reconstructive Surgery, Juntendo University Graduate School of Medicine, 2-1-1, Hongo, Bunkyo-ku, Tokyo 113-8421, Japan
[10]Department of Signal Transduction, Research Institute for Microbial Diseases, Osaka University, 3-1 Yamada-oka, Suita, Osaka 565-0871, Japan
[11]Laboratory of Signal Transduction, World Premier Institute Immunology Frontier Research Center, Osaka University, 3-1 Yamada-oka, Suita, Osaka 565-0871, Japan
[12]Lead contact
*Correspondence: ntakaku@biken.osaka-u.ac.jp (N.T.), naito.hisamichi@gmail.com (H.N.)
https://doi.org/10.1016/j.stemcr.2026.102931

SUMMARY

Endothelial cells expressing the CD157 antigen (CD157$^+$ ECs) contribute to vascular regeneration and maintenance in adult tissues, but their molecular identity is not fully defined. Here, we show that *CD157*-positive ECs in mouse and human tissues share conserved transcriptional profiles enriched for angiogenesis-associated genes. Regulon analysis revealed a gene-regulatory network in which the NFAT pathway contributes to vascular network formation. Integration of mouse and human scRNA-seq datasets revealed human EC clusters with gene expression profiles resembling mouse *CD157*-positive ECs. The clusters were localized in the large vessel intima and expressed known stem-like EC markers such as *BST1* (*CD157*), *PROCR* (*CD201*), and *ABCG2*. Functionally, human CD157$^+$ ECs isolated from muscle exhibited greater proliferative capacity than CD157$^-$ ECs. Cell-cell interaction analysis suggested active communication between *CD157*-positive ECs and surrounding cells, via the CXCL12-CXCR7 axis. Our findings identify a conserved gene signature for CD157$^+$ ECs with potential relevance for vascular regeneration.

INTRODUCTION

Blood vessels form an essential systemic network that maintains homeostasis through the delivery of oxygen and nutrients to tissues and the removal of metabolic waste. Endothelial cells (ECs), which line the inner surface of blood vessels, play an essential role in these functions. Under hypoxic conditions, ECs become activated and initiate angiogenesis (the formation of new blood vessels from pre-existing ones) to restore oxygen supply (Potente et al., 2011). This process is tightly regulated by signaling pathways such as VEGF and Notch and involves dynamic changes in EC states (Blanco and Gerhardt, 2013).

Several research groups, including ours, have recently proposed that peripheral blood vessels may contain EC stem/progenitor cell populations that contribute to angiogenesis and vascular regeneration, particularly following severe endothelial injury (Chambers et al., 2021; Naito et al., 2020a). We previously identified CD157 (Bst1) as a cell surface marker that distinguishes stem cell-like ECs (also known as vascular endothelial stem cells [VESCs]) from other ECs (Wakabayashi et al., 2018). CD157, a glycosylphosphatidylinositol-anchored membrane protein with ADP-ribosyl cyclase activity, is involved in NAD$^+$ metabolism, generating cyclic ADP-ribose and releasing nicotinamide. It is mainly expressed in bone marrow stromal and certain myeloid cell populations and has functions in cell adhesion, migration, and immune regulation (Ortolan et al., 2019). However, its expression in vascular ECs is not widely recognized. Paneth cells in the intestinal stem cell niche also express CD157, suggesting a potential role in regulating stem cell microenvironments (Yilmaz et al., 2012). Nevertheless, the precise characteristics of CD157$^+$ VESCs and the extent of their conservation across different organs remain unclear, and are even less well understood in humans.

One approach that may provide a deeper understanding of the VESC characteristics is single-cell RNA sequencing

Published by Elsevier Inc. on behalf of International Society for Stem Cell Research.
This is an open access article under the CC BY license (http://creativecommons.org/licenses/by/4.0/).

(scRNA-seq). In vascular biology, scRNA-seq is now widely used to analyze endothelial heterogeneity under physiological and pathological conditions (Kalucka et al., 2020; Pan et al., 2024). Yet, few scRNA-seq analyses have focused specifically on EC stem/progenitor populations.

Here, we performed scRNA-seq analyses of ECs isolated from adult mouse liver, skeletal muscle, and lung, as well as from adult human skeletal muscle, focusing on *CD157*-positive ECs. In mice, *CD157*-positive ECs were enriched in a venous-like cluster and shared a gene expression signature associated with angiogenesis. Regulon analysis suggested involvement of the nuclear factor of activated T-cells (NFAT) pathway. Fresh human lower limb muscle samples showed an EC subcluster with a gene expression profile similar to that of mouse CD157$^+$ ECs. Human CD157$^+$ ECs had a higher proliferative capacity and were primarily localized to large vessels. Integration of publicly available scRNA-seq datasets suggested conserved intercellular communication patterns between *CD157*-positive ECs and neighboring cells. These findings suggest that CD157$^+$ ECs share common transcriptional and functional features across tissues and species and may localize to specific vascular niches.

RESULTS

CD157-positive ECs reside in a specific cluster with conserved gene expression across tissues in mice

We investigated the gene expression profiles of CD157$^+$ ECs in different tissues using fluorescence-activated cell sorting (FACS) to isolate CD31$^+$CD45$^-$ EC fractions from mouse liver, hindlimb muscle, and lung, followed by scRNA-seq analysis of these populations (Figures 1A and 1B). Vascular ECs were distinguished from contaminating cells by the expression of well-established cell type markers. Cells expressing *Cdh5*, *Pecam1* (*CD31*), and *Emcn*, and negative for *Ptprc* (*CD45*) and *Prox1* were classified as vascular ECs (Trimm and Red-Horse, 2023; Wakabayashi and Naito, 2023).

In the liver, 12,972 cells were initially detected. Lymphatic ECs (*Prox1* and *Pdpn*), pericyte/smooth muscle cells (SMCs) (*Pdgfrb* and *Acta2*), cholangiocytes (*Krt8*), and hepatocytes (*Alb*) were excluded from further analysis. As a result, 11,739 liver ECs were analyzed, and unbiased clustering identified nine clusters. We validated the cluster identity of vascular ECs using canonical marker genes and the top 10 differentially expressed genes (DEGs) and classified the vascular ECs into two portal vein/hepatic artery EC clusters (clusters 0 and 1 marked by *Cd34*, *Sox17*, *Gja5*, and *Ly6a*), one zone 1 liver sinusoidal EC cluster (cluster 2 marked by *Ntn4* and *Msr1*), three zone 2 liver sinusoidal EC clusters (clusters 3, 4, and 5 marked by *Stab1*, *Stab2*, and *Mrc1*), one zone 3 liver sinusoidal EC cluster (cluster 6 marked by *Cdh13*, *Thbd*, and *Fabp4*), one central vein EC cluster (cluster 7 marked by *Wnt2*, *Wnt9b*, and *Rspo3*), and one proliferating EC cluster (cluster 8 marked by *Mki67* and *Top2a*) (Figures 1C, 1D, and S1A) (Su et al., 2021).

In the hindlimb muscle, 11,989 cells were initially detected. Lymphatic ECs (*Prox1* and *Pdpn*), pericytes/SMCs (*Acta2* and *Pdgfrb*), fibroblasts (*Fn1* and *Col1a1*), muscle cells (*Actn3*, Des, and *Tnnc2*), and Schwann cells (*Sox10*, *Mpz*, and *Mbp*) were removed, leaving 9,220 ECs for analysis. Unbiased clustering of vascular ECs identified seven clusters classified into one arterial EC cluster (cluster 0 marked by *Efnb2*, *Gja4*, and *Gja5*), five capillary EC clusters (clusters 1, 2, 3, 4, and 5 marked by *Rgcc* and *Sparc*), and one venous EC cluster (cluster 6 marked by *Nr2f2*, *Ephb4*, and *Ackr1*) (Figures 1C, 1D, and S1B).

In the lung, 7,911 cells were initially detected. Lymphatic ECs (*Prox1* and *Pdpn*), type II epithelial cells (*Sftpc*, Sftpa1, and *S100g*), type I epithelial cells (*Akap5*, *Hopx*, and *Cav1*), blood cells (*Ptprc*), fibroblasts (*Fn1* and *Col1a1*), and pericytes/SMCs (*Pdgfrb* and *Acta2*) were excluded from downstream analyses, leaving 6,636 ECs from the lung for analysis. Unbiased clustering of vascular ECs identified six clusters classified into one arterial EC cluster (cluster 0 marked by *Efnb2*, *Gja4*, and *Gja5*), three capillary EC clusters (clusters 1, 2, and 3 marked by *Rgcc* and *Sparc*), one venous EC cluster (cluster 4 marked by *Nr2f2*, *Ephb4*, and *Slc6a2*), and one aerocyte (aCap) cluster (cluster 5 marked by *Car4*) (Figures 1C, 1D, and S1C).

Based on the classification of the single-cell data, analysis of the distribution of *CD157*-positive cells across EC subpopulations showed that they accounted for 2.0% of total vascular ECs in the liver, 3.9% in the limb muscle, and 8.0% in the lung (Figure 1E). In the liver, these cells were most abundant in the central and portal vein clusters, with fewer detected in the hepatic artery cluster. In the limb muscle and lung, they localized predominantly to venous-like EC clusters. These findings suggest a preferential association of *CD157*-positive ECs with larger vessel regions rather than capillaries. Consistently, immunofluorescence staining of mouse tissues confirmed their localization to the endothelium of relatively large vessels in the liver, muscle, and lung (Figure 1F).

We next examined the common features of the *CD157*-positive ECs in each tissue by analyzing highly expressed genes in these cells. Compared to other ECs, the *CD157*-positive ECs in large vessel regions (clusters 0, 1, and 7 in the liver, cluster 6 in the hindlimb, and cluster 4 in the lung) exhibited differential expression profiles, defined by a fold-change threshold >1.5 for upregulated and <0.667 for downregulated genes, with 203 upregulated and 230 downregulated genes in the liver, 253 and 183 in the

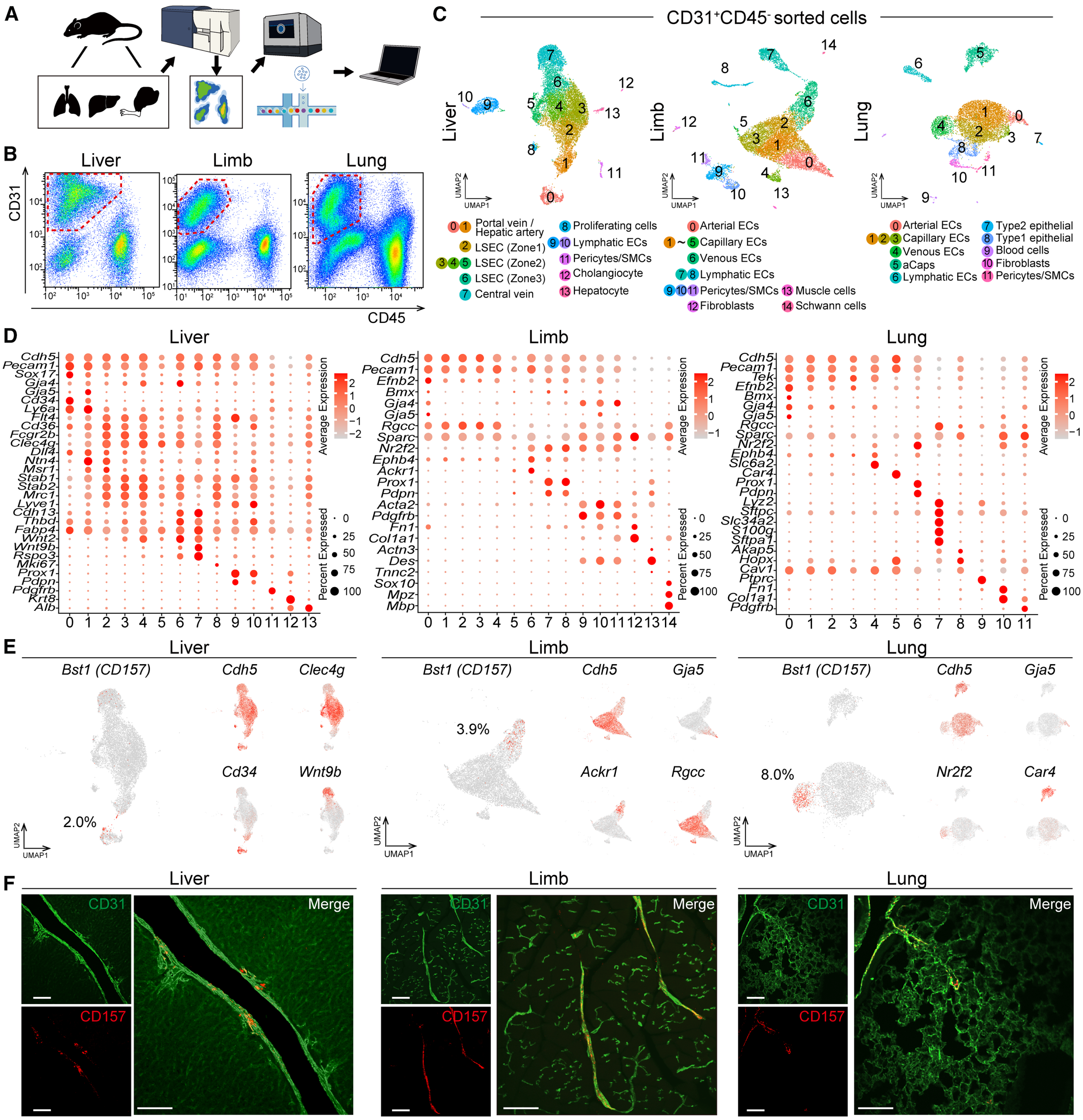

Figure 1. Single-cell RNA-seq identifies *CD157*-positive ECs in the mouse vasculature of liver, muscle, and lung
(A) Schematic diagram showing the experimental workflow.
(B) Representative flow cytometry dot plots of cells obtained from mouse liver, hindlimb muscle, and lung. The red gate indicates CD31^{+}CD45^{-} cells isolated for single-cell analysis.
(C) UMAP plots of scRNA-seq data from sorted cells isolated from mouse liver (12,972 cells), hindlimb muscle (11,989 cells), and lung (7,911 cells).
(D) Bubble plots comparing the expression levels of selected signature genes across clusters of CD31^{+}CD45^{-} cells.
(E) Feature plots of tissue-specific EC markers projected onto UMAP plots of CD31^{+}CD45^{-} sorted cells.
(F) Immunofluorescence images of the mouse liver, hindlimb muscle, and lung stained for CD31 (green) and CD157 (red). Single-channel images are shown on the left. Scale bars, 100 μm.

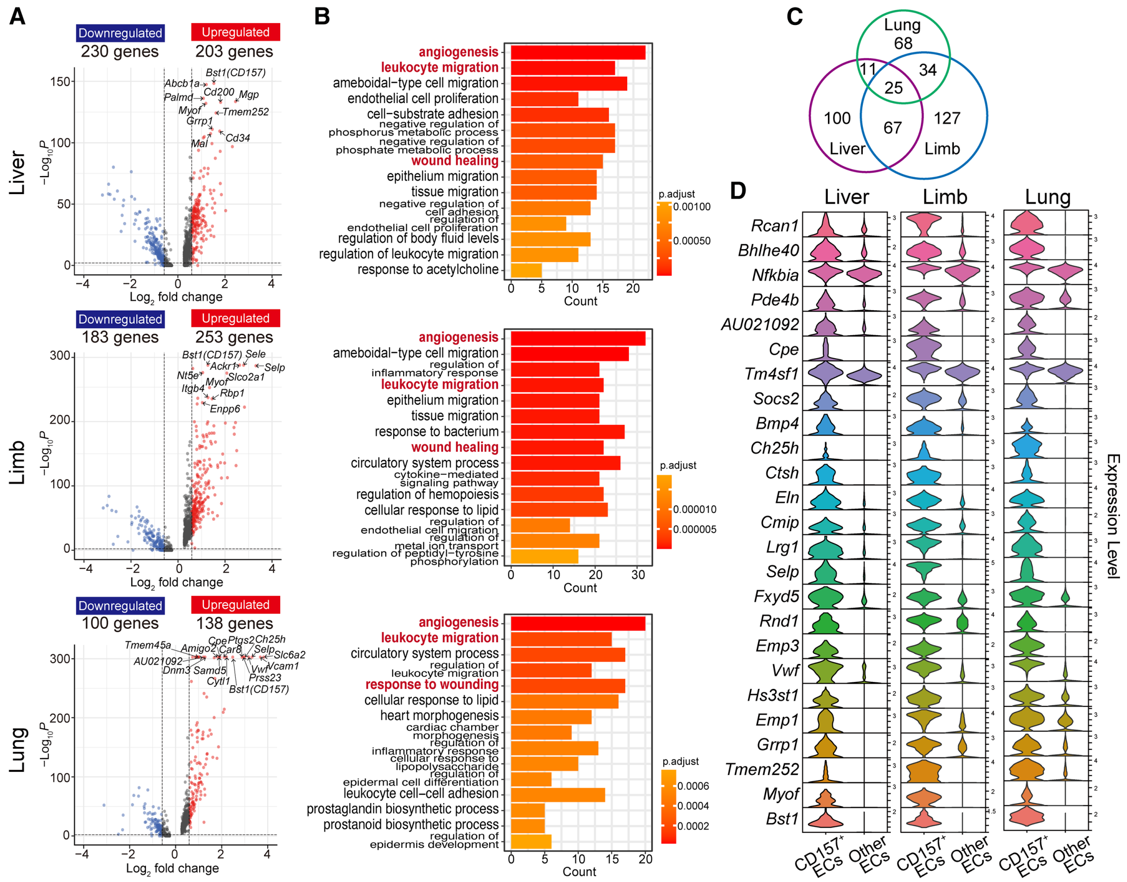

Figure 2. *CD157*-positive ECs from different mouse tissues share common gene expression profiles and pathways

(A) Volcano plots showing gene expression changes between *CD157*-positive ECs in venous clusters and other ECs in each indicated tissue. Cutoff values were set at a fold change of 1.5 and a *p* value of 0.01. The numbers below the "upregulated genes" and "downregulated genes" labels indicate the number of genes exceeding these thresholds. Dots labeled with gene names represent the top 10 genes most highly expressed in *CD157*-positive ECs.

(B) GO pathway analysis of genes characteristically upregulated in *CD157*-positive ECs in each tissue. GO analysis was performed using the upregulated genes identified in Figure 2A.

(C) Venn diagram of upregulated genes detected in the three indicated tissues.

(D) Violin plots comparing *CD157*-positive ECs in venous clusters with remaining ECs, showing the expression of 25 upregulated genes shared across the three tissues.

hindlimb muscle, and 138 and 100 in the lung (Figure 2A). Gene ontology (GO) analysis of the biological processes associated with these gene sets revealed enrichment of genes in pathways related to angiogenesis, wound healing, and leukocyte migration in all three tissues (Figure 2B). Across tissues, we identified 25 common DEGs that were highly expressed in *CD157*-positive ECs. Although without statistical significance, we found potential enrichment signals implicating VEGF receptor signaling pathways (Figures 2C, 2D, and S2). These results suggest shared gene expression profiles in *CD157*-positive cells in all three tissues and conserved angiogenic features regardless of tissue origin.

CD157-positive ECs exhibit transcriptomic commonality across tissues and aggregate into a conserved vascular subpopulation

Previous scRNA-seq studies have shown marked heterogeneity and tissue-specific gene expression in vascular ECs. Integration of single-cell datasets across tissues typically reveals few shared clusters (Kalucka et al., 2020). Reanalysis of publicly available mouse scRNA-seq datasets revealed scarce numbers of *CD157*-positive ECs in the liver, brain, and heart under physiological conditions, whereas they were abundant in the lung and ischemic heart (Figure S3) (Li et al., 2019, 2024; Profaci et al., 2024; Rodor et al., 2022; Su et al., 2021). In contrast, our tissue-specific

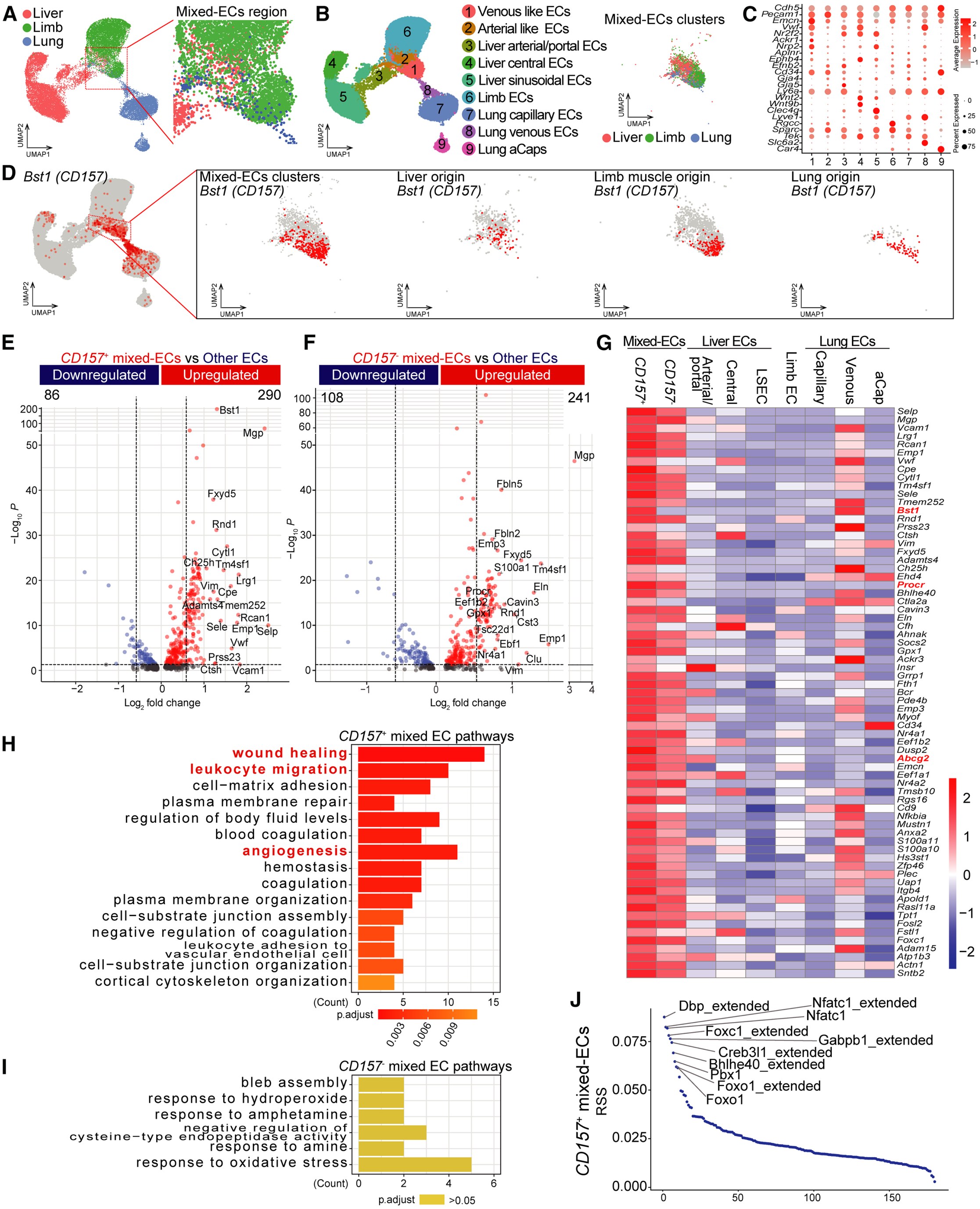

Figure 3. *CD157*-positive ECs are enriched in a mixed-EC cluster and share common gene expression profiles in mice

(A) UMAP plot of 27,595 ECs integrated from three mouse tissues, color-coded by tissue of origin. The region outlined by the red box indicates an area where cells from all three tissues are intermixed.

(B) UMAP plots of integrated ECs. The right image shows a magnified view of a cluster composed of cells derived from all three tissues.

(C) Bubble plots comparing the expression levels of selected signature genes across clusters defined in Figure 3B.

(D) Feature plots of *Bst1 (CD157)* expression projected onto the UMAP plots of integrated ECs. The feature plots within the black box on the right show *Bst1* expression in mixed-EC cluster, followed by separate projections of *Bst1* expression onto the UMAPs of the integrated dataset, split according to tissue.

(legend continued on next page)

analyses in mice indicated that *CD157*-positive cells represent a conserved EC subpopulation. Therefore, we investigated whether our dataset, which included a larger number of these ECs, contained subpopulations shared across multiple tissues.

Unbiased integration of single-cell data from each tissue, comprising 27,595 ECs in total, revealed tissue specificity for most ECs, but a population composed of cells derived from all tissues was also present (Figure 3A). Cluster classification of the integrated dataset showed that this mixed-origin EC population (hereafter referred to as mixed-ECs) formed distinct clusters characterized by venous and arterial-like endothelial features (Figures 3B and 3C). The remaining ECs were classified according to their tissue origin. ECs of the liver were classified into a central vein EC cluster (*Wnt2* and *Wnt9b*), liver sinusoidal EC cluster (*Lyve1* and *Robo4*), and arterial/portal vein EC cluster (*Cd34*, *Gja5*, and *Ly6a*). The limb muscle ECs were classified as a capillary EC cluster (*Rgcc*). The lung ECs were classified into a capillary EC cluster (*Sparc* and *Tek*), venous EC cluster (*Slc6a2*), and aCap cluster (*Car4*) (Figures 3C and S4A). This integrated clustering framework revealed a predominance of the *CD157*-positive ECs within these mixed-EC clusters (Figure 3D). However, the *CD157*-positive ECs from the lung were not restricted to the mixed-EC clusters, but were also broadly distributed within the lung venous cluster and, to a lesser extent, within the capillary EC cluster.

Comparison of the characteristics of *CD157*-positive ECs in the mixed-EC clusters to those of all other vascular ECs identified 290 upregulated and 86 downregulated genes (Figure 3E). A similar comparison of *CD157*-negative ECs in the mixed-EC clusters and other vascular ECs identified 241 upregulated and 108 downregulated genes (Figure 3F). Of these, 69 genes upregulated in *CD157*-positive ECs and 30 in *CD157*-negative ECs exhibited fold changes >1.5. Heatmap analysis confirmed high expression of these genes in the mixed-EC cluster. Notably, *Procr* and *Abcg2*, both recognized as VESC markers (Lin et al., 2024; Yu et al., 2016), were upregulated in the *CD157*-positive mixed-ECs (Procr; FC 1.97, Abcg2; FC 1.73) (Figure 3G). Pathway enrichment analysis revealed associations with angiogenesis, wound healing, and leukocyte migration (Figures 3H and S4B). Comparison of *CD157*-positive and *CD157*-negative cells within the mixed-EC population showed higher expression of most of these pathway-associated genes in *CD157*-positive cells (Figures S4C and S4D). No specific pathways were identified for the upregulated genes in *CD157*-negative ECs (Figure 3I). Regulon analysis of *CD157*-positive ECs identified several key transcriptional regulators involved in vascular regulation, including *Nfatc1*, *Foxc1*, and *Creb3l1* (Figure 3J) (Binet and Sapieha, 2015; Seo et al., 2006; Suehiro et al., 2014). These findings suggested that *CD157*-positive ECs are enriched within mixed-EC clusters and may share a common vascular regulatory system.

The NFAT pathway is essential for CD157$^+$ EC network formation

To validate these findings, and because Procr is a known VESC marker, we examined CD157 and Procr expression by FACS and identified four EC populations across all three tissues: CD157$^+$Procr$^-$, CD157$^-$Procr$^+$, CD157$^-$Procr$^-$, and CD157$^+$Procr$^+$ (Figure 4A). Immunofluorescence staining confirmed CD157 and Procr protein expression patterns consistent with the FACS analysis in all tissues (Figure 4B). Culture of liver-derived fractions showed that CD157$^+$Procr$^+$ ECs had the highest proliferative capacity (Figures 4C and 4D).

Given the enrichment of NFAT-related transcriptional activity in *CD157*-positive ECs, we next examined the role of NFAT in these populations by inhibiting the NFAT pathway in each EC subpopulation with cyclosporin A (CsA). Although CsA suppressed network formation in all EC subpopulations, the reduction in network area was significantly greater in CD157$^+$Procr$^+$ and CD157$^+$Procr$^-$ ECs than in the other two fractions

(E) Volcano plots showing gene expression changes between *CD157*-positive cells within the mixed-origin cluster and all other ECs. Genes with p values below 0.05 were considered significant; red dots indicate upregulated genes in *CD157*-positive cells, and blue dots indicate downregulated genes. The numbers below the "upregulated genes" and "downregulated genes" labels indicate the number of significantly changed genes. Horizontal line: p = 0.05; vertical lines: fold-change >1.5 (upregulated) or <0.67 (downregulated).

(F) Volcano plots showing gene expression changes between *CD157*-negative cells within the mixed-origin cluster and all other ECs. Genes with p-values below 0.05 were considered significant; red dots indicate upregulated genes in *CD157*-negative cells, and blue dots indicate downregulated genes. The numbers below the 'Upregulated Genes' and 'Downregulated Genes' labels indicate the number of significantly changed genes. Horizontal line: p = 0.05; vertical lines: fold-change >1.5 (upregulated) or <0.67 (downregulated).

(G) Heatmap showing the expression of genes that met the fold-change cutoff of >1.5 detected in Figure 3E.

(H) GO pathway analysis of upregulated genes in *CD157*-positive ECs within the mixed-EC cluster.

(I) GO pathway analysis of upregulated genes in *CD157*-negative ECs within the mixed-EC cluster.

(J) Regulon specificity score (RSS) plots showing the ranking of regulons specifically active in *CD157*-positive ECs within the mixed-EC cluster.

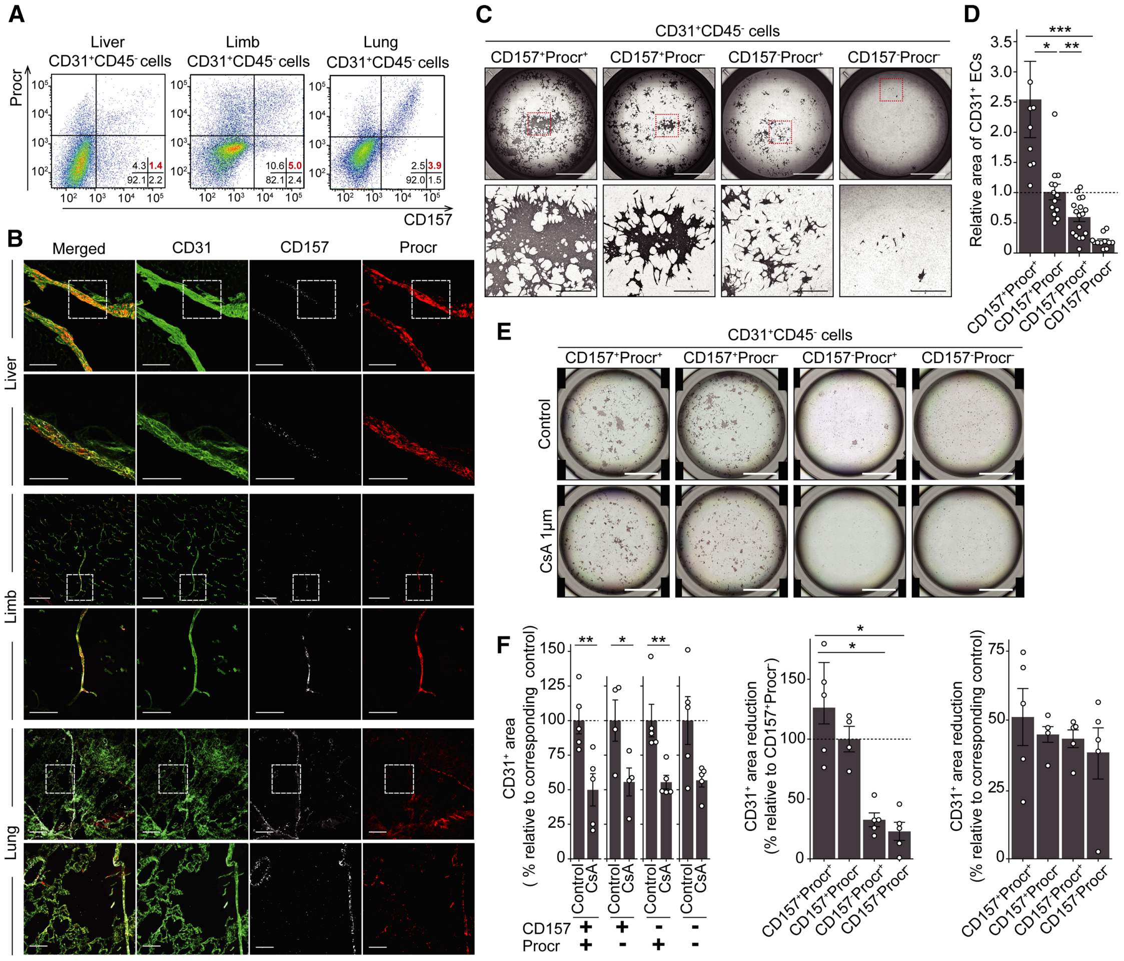

Figure 4. Mouse CD157^{+}Procr^{+} ECs exhibit high proliferative potential and NFAT contributes to vascular network formation

(A) Dot plots showing CD31^{+}CD45^{-} cells from the mouse liver, hindlimb muscle, and lung analyzed for CD157 and Procr expression.

(B) Immunofluorescence images of mouse liver, hindlimb muscle, and lung stained for CD31 (green), CD157 (white), and Procr (red). Enlarged views of the areas outlined with white dashed lines in the upper images are shown in the lower images. Scale bars, 100 μm (upper) and 50 μm (lower).

(C) Endothelial colony formation assay using 3,000 mouse liver-derived CD157^{+}Procr^{+}, CD157^{+}Procr^{-}, CD157^{-}Procr^{+}, and CD157^{-}Procr^{-} ECs cultured on OP9 feeder cells. Cells were stained with anti-CD31 antibody. Enlarged views of the areas outlined with red dashed lines in the upper images are shown in the lower images. Scale bars, 5 mm (upper) and 1 mm (lower).

(D) Bar plots showing the relative quantification of CD31^{+} areas formed by each EC subpopulation after 10 days of culture, as shown in Figure 4C. Dots represent different wells ($n > 8$). Data from five independent isolation experiments. Data are presented as mean ± SEM. ***$p < 0.0005$, **$p < 0.005$, *$p < 0.05$.

(E) Endothelial colony formation under CsA treatment (1 μM), visualized by anti-CD31 immunostaining, using mouse liver-derived CD157^{+}Procr^{+}, CD157^{+}Procr^{-}, CD157^{-}Procr^{+}, and CD157^{-}Procr^{-} ECs. Scale bars,sss 5 mm.

(F) Bar plots showing, from left to right, CD31^{+} area relative to the corresponding control; reduction in CD31^{+} area normalized to the reduction observed in CD157^{+}Procr^{-} cells; and reduction in CD31^{+} area relative to the corresponding control, after 10 days of culture with or without CsA, as shown in Figure 4E. Dots represent individual wells ($n > 3$). Data are derived from three independent experiments. Data are presented as mean ± SEM. **$p < 0.005$, *$p < 0.05$.

(Figures 4E and 4F). These results suggest that inhibiting the highly proliferative CD157^{+} population results in a more pronounced reduction in vascular network formation, while also supporting a broader role for NFAT signaling across EC fractions.

Identification of stem-like vascular ECs in humans

VESCs have rarely been characterized in humans, and their defining features are largely unknown. To identify and characterize these cells in humans, we isolated CD31^{+}CD45^{-} cells from human lower limb muscle and

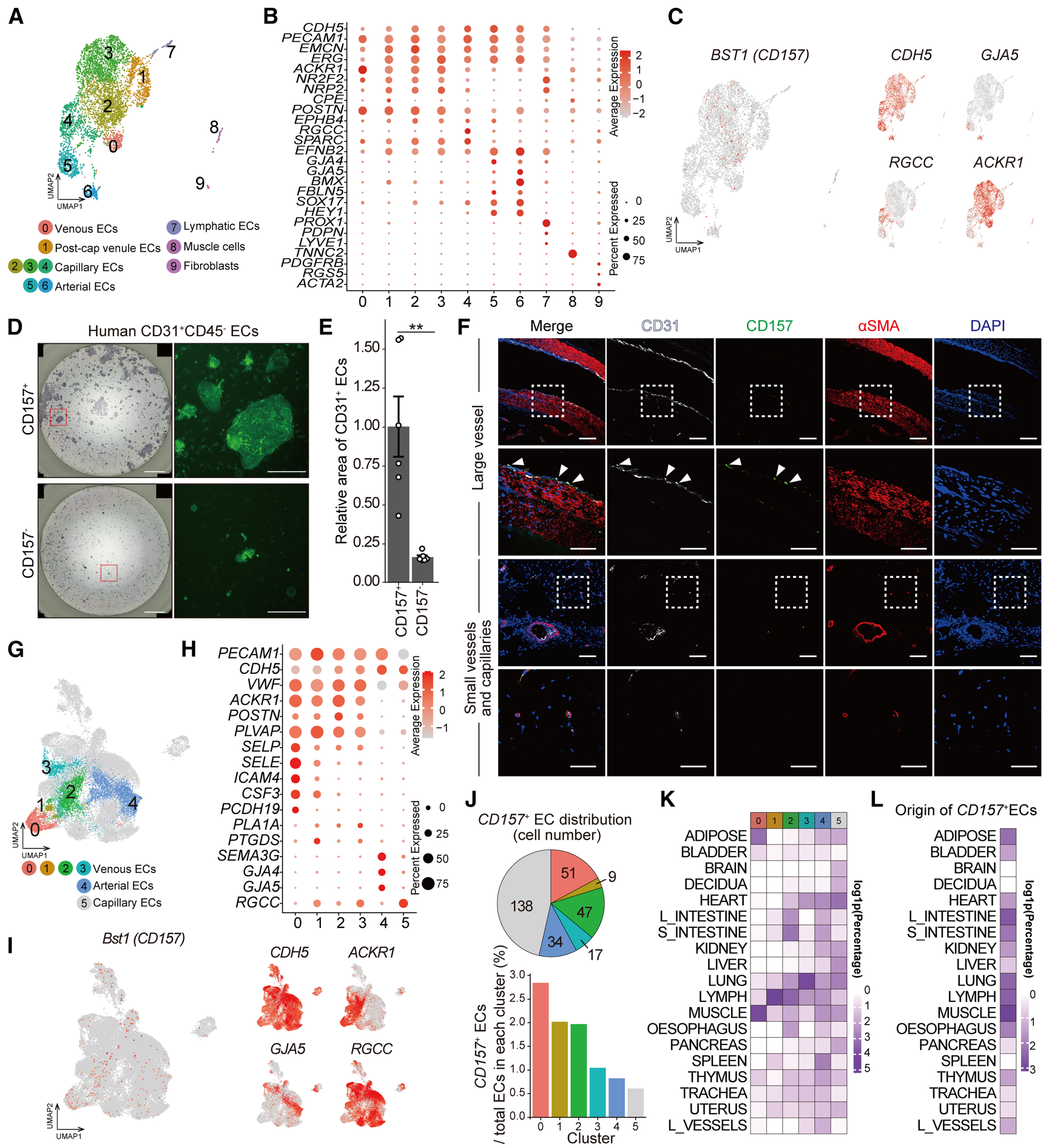

Figure 5. Identification of a human VESC-enriched cluster

(A) UMAP plots of scRNA-seq data from sorted $CD31^+CD45^-$ cells isolated from human lower limb muscle (4,958 cells).

(B) Bubble plots comparing the expression levels of selected signature genes across clusters in the $CD31^+CD45^-$ cells isolated from human lower limb muscle.

(C) Feature plots of *CD157* (*BST1*) and arterial, capillary, and venous EC markers projected onto the UMAP shown in Figure 5A.

(D) Endothelial colony formation assay using 5,000 human lower limb-derived $CD157^+$ and $CD157^-$ ECs cultured on OP9 feeder cells. Cells were stained with anti-CD31 antibody. Enlarged views of the areas outlined by red dashed lines in the left images are shown in the right images. Scale bars, 1 mm (left) and 200 µm (rightss).

(E) Bar plots showing the relative quantification of $CD31^+$ areas formed by $CD157^+$ and $CD157^-$ ECs after 10 days of culture, as shown in Figure 5D. Dots represent individual areas ($n = 6$). Data are derived from two independent experiments. Data are presented as mean ± SEM. $**p < 0.005$.

(F) Immunofluorescence images of human soleus muscle showing large vessels and small vessels/capillaries stained for CD31 (white), CD157 (green), αSMA (red), and DAPI (blue). Enlarged views of the regions outlined by white dashed lines in the upper images are shown in the lower images. Arrows indicate $CD157^+$ ECs. Scale bars, 100 µm (upper) and 50 µm (enlarged lower images).

(legend continued on next page)

performed scRNA-seq analysis. Unbiased clustering of the 4,958 sorted cells revealed 10 clusters (Figures 5A and 5B). Based on the expression of well-established cell type markers, cells expressing *CDH5*, *PECAM1* (*CD31*), and *EMCN* but negative for *PROX1* were classified as vascular ECs, whereas lymphatic ECs (*PROX1*, *PDPN*, and *LYVE1*), muscle cells (*TNNC2*), and pericytes/SMCs (*PDGFRB*, *RGS5*, and *ACTA2*) were identified as non-EC populations. As a result, 4,779 vascular ECs were identified and further classified into seven EC clusters. In this dataset, a small number of *CD157*-expressing cells was detected in capillary and venous EC populations (Figure 5C). Assessment of interindividual variability using scRNA-seq analysis of ECs isolated from an independent human lower limb muscle sample revealed a similar distribution pattern in this independent dataset (Figures S5A and S5B).

We next isolated $CD31^{+}CD45^{-}CD157^{+}$ ECs from human lower limb muscle to evaluate their proliferative capacity (Figure S5C). The proliferative capacity was higher in these cells than in $CD157^{-}$ ECs, and they formed colony-like clusters resembling those formed by mouse $CD157^{+}$ ECs (Figures 5D and 5E) (Wakabayashi et al., 2018). $CD157^{+}$ ECs were detected in larger vessels surrounded by multilayered αSMA-positive SMCs, whereas CD157 was not detected in capillary ECs, including capillaries covered by a αSMA-positive cell monolayer (Figure 5F). Therefore, although $CD157^{+}$ ECs in the human dataset did not form the clearly segregated cluster observed in mouse tissues, the functional and anatomical data showed similarities between both species. Reanalysis of publicly available multi-organ EC scRNA-seq datasets also identified a subset of $CD157^{+}$ ECs enriched within venous clusters, consistent with our findings in mice (Figures 5G–5K) and originating from multiple organs (Figure 5L) (Barnett et al., 2024).

Comparative scRNA-seq analysis of mouse and human ECs indicates conservation of *CD157*-positive ECs

Based on these findings, we hypothesized that stem-like ECs exist in the human vasculature. We performed cross-species integration to characterize human *CD157*-positive ECs and assess their conservation with mouse *CD157*-positive ECs. Recognizing that integration between mouse and human datasets is often challenging and does not consistently achieve the same level of alignment obtained in mouse-to-mouse dataset integration, we integrated the mouse and human datasets by converting mouse gene symbols to their human orthologs. We selected only the genes common to both species and applied multiple integration methods for clustering (Figure S6A). Among the methods tested, Harmony integration yielded the most balanced mixing of mouse and human cells while preserving arterial, venous, and capillary classifications (Figure S6B). Therefore, this method was used for the downstream analyses. The Harmony-integrated data were classified into 18 clusters (Figures 6A and 6B) and further classified into three venous-like EC clusters (clusters 0, 1, and 2 marked by *ACKR1* and *NR2F2*), two post-capillary venule clusters (clusters 3 and 4 marked by *POSTN*), five capillary clusters (clusters 5–9 marked by *RGCC* and *SPARC*), two arterial-like EC clusters (clusters 10 and 11 marked by *EFNB2*, *GJA4*, and *GJA5*), two lymphatic EC clusters (clusters 12 and 13 marked by *PROX1*, *PDPN*, and *LYVE1*), two pericyte/SMCs clusters (clusters 14 and 15 marked by *PDGFRB*, *RGS5*, and *ACTA2*), one muscle cell cluster (cluster 16 marked by *TNNC2*), and one fibroblast cluster (cluster 17 marked by *FN1* and *COL1A1*). In this integrated dataset, both mouse and human *CD157*-positive cells accumulated in clusters 0, 1, and 2, although their numbers were limited in the human dataset (Figures 6C, 6D, and S6C). Despite their limited number, the *CD157*-positive cells accumulated within the venous EC cluster after integration. *PROCR*- and *ABCG2*-positive cells were also predominantly enriched in the same clusters; however, they were also detected to some extent in other clusters. Likewise, *NFATC1* was widely expressed but exhibited approximately twice the expression frequency in these clusters than in the other clusters.

To account for potential bias arising from differences in cell numbers between human and mouse datasets, we also performed cross-species integration of mouse hindlimb cells (12,681 cells) with $CD31^{+}CD45^{-}$ cells from one independent human sample (12,915 cells; UMAP shown in Figure S5A) and, separately, with two human samples (17,873 cells; UMAP shown in Figure S5B). Again, a subset of human *CD157*-positive ECs co-localized within clusters enriched for mouse *CD157*-positive ECs (Figures S6D and S6E). Another subset of human *CD157*-positive ECs localized to a venous/capillary-like cluster characterized by *ACKR1* expression, but this was not detected in the mouse dataset.

(G) UMAP plots from reanalysis of publicly available human EC scRNA-seq datasets.
(H) Bubble plots comparing the expression levels of selected signature genes across clusters in Figure 5G.
(I) Feature plots of *CD157* (*BST1*) and arterial, capillary, and venous EC markers projected onto the UMAP shown in Figure 5G.
(J) Pie chart showing the number of *CD157*-positive cells in each cluster in Figure 5G, and bar graph showing the proportion of *CD157*-positive cells in each cluster.
(K) Heatmap representation of cluster enrichment per tissue in Figure 5G.
(L) Proportion of *CD157*-positive cells by tissue of origin.

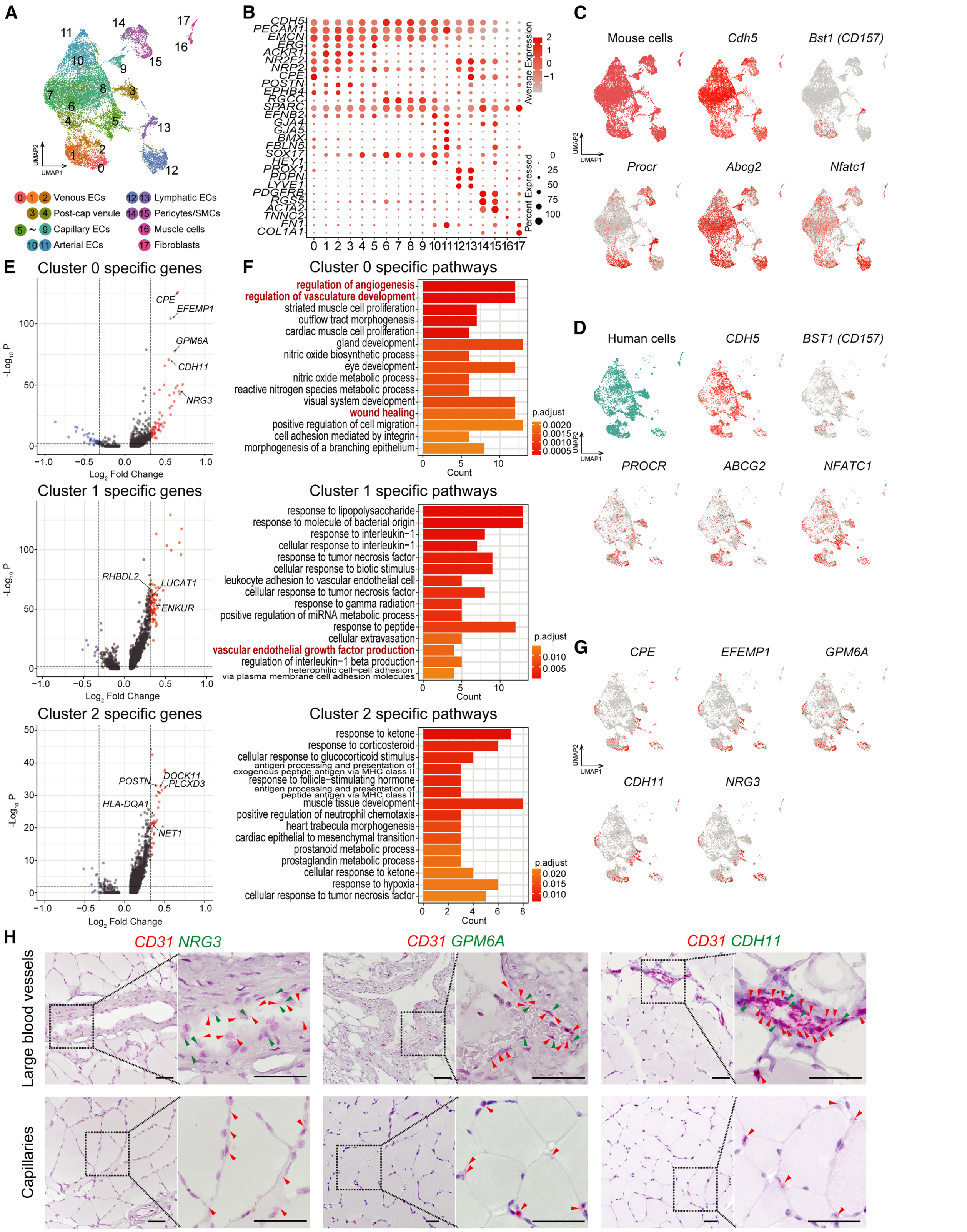

(legend on next page)

To minimize complexity introduced by multi-sample integration and batch correction, we performed downstream analyses using an integrated dataset comprising a single human sample and the mouse dataset and evaluated the gene expression signatures of clusters containing *CD157*-positive ECs (Figure 6E; Table S1). Comparison with the mouse *CD157*-positive EC markers defined in Figure 3G revealed limited overlap between the three clusters (Figure S6F), suggesting modest direct gene-level similarity. However, examination of cluster-specific pathways revealed that cluster 0 was enriched for angiogenesis-, vasculogenesis-, and wound healing-related pathways, resembling the enrichment observed in the mouse *CD157*-positive EC population (Figure 6F). Therefore, we focused on cluster 0 and confirmed that its top five signature genes (*CPE*, *EFEMP1*, *GPM6A*, *CDH11*, and *NRG3*) were predominantly expressed in this cluster (Figure 6G). Further verification of the expression patterns of these genes in mice datasets revealed a partial co-expression of *Cpe*, *Efemp1*, and *Gpm6a* with *CD157* in limb muscle ECs and in the integrated dataset. However, *Cdh11* was scarcely expressed in the capillary cluster, and *Nrg3* was not detected in either dataset (Figure S6G).

Although not all genes expressed in cluster 0 overlapped with those in the mouse *CD157*-positive cluster, we investigated the spatial distribution of ECs of this cluster by performing *in situ* hybridization of human lower limb muscle tissue for *NRG3*, *GPM6A*, and *CDH11*. These genes were not detected in capillaries but were expressed in ECs associated with relatively large vessels, consistent with the distribution of $CD157^+$ ECs (Figure 6H). These findings suggest the presence of human ECs with gene expression profiles partially resembling those of mouse $CD157^+$ VESCs and support the existence of a conserved subpopulation localized to large blood vessels.

Bioinformatic analysis reveals interactions between *CD157*-positive ECs and pericytes/SMCs in mouse

We further performed bioinformatic analysis to evaluate potential interactions between *CD157*-positive ECs and surrounding cells. Stem-like cells are known to form niches within tissues and to engage in distinct cell–cell interactions with surrounding cells (Hsu and Fuchs, 2012). We explored the microenvironment associated with *CD157*-positive ECs by collecting publicly available mouse scRNA-seq datasets encompassing all cell types from the liver, limb, and lung (Reyfman et al., 2019; Schaum et al., 2018; Xiong et al., 2019). We then integrated these whole-tissue datasets (48,972 cells) with our vascular EC dataset (32,872 cells), totaling 81,844 cells, to analyze cell-cell interactions between *CD157*-positive ECs and surrounding cell types. The vascular EC population clustered in a manner consistent with our EC-only dataset, including a mixed-EC cluster, while the surrounding cells clustered according to their respective cell types (Figures 7A–7C). Cell-cell interaction analysis using CellPhoneDB revealed that *CD157*-positive ECs within the mixed-EC cluster engaged in numerous interactions with surrounding cells, particularly pericytes/SMCs (Figure 7D). Given that these interactions exhibited the highest number of signaling events, we also used CellChat to analyze pericyte/SMC-derived signals toward *CD157*-positive ECs. The predominant signals were associated with the extracellular matrix (i.e., collagen and laminin), followed by CXCL signaling (Figure 7E).

CXCL signaling plays key roles in angiogenesis, cell migration, and inflammatory cell recruitment (Mehrad et al., 2007). Based on communication probability scores, CXCL signaling activity was approximately 2-fold higher between *CD157*-positive ECs in the mixed-EC cluster and pericytes/SMCs than in other EC clusters (Figure 7F; Table S2). The

Figure 6. Comparative scRNA-seq analysis of mouse and human ECs reveals a conserved CD157-enriched EC cluster and candidate human markers

(A) UMAP of integrated dataset combining human lower limb muscle $CD31^+CD45^-$ cells (4,958 cells) and mouse hindlimb muscle $CD31^+CD45^-$ cells (12,681 cells), totaling 17,639 cells.

(B) Bubble plots comparing the expression levels of selected signature genes across cell clusters from the integrated dataset shown in Figure 6A.

(C and D) Feature plots of VESC-associated genes projected onto species-split UMAPs of the integrated dataset: mouse (C) and human (D).

(E) Volcano plots showing DEGs for the human-derived components of clusters 0, 1, and 2 from the integrated dataset. Genes with a p value < 0.05 and a fold change (FC) > 1.25 were considered significant. Among the upregulated genes, the top five with the highest fold change and strong cluster specificity (defined as expression in <20% of cells outside the target cluster) are shown. If fewer than five genes met these criteria, all qualifying genes are displayed.

(F) Results of GO enrichment analysis for DEGs identified in clusters 0, 1, and 2 from the integrated dataset (p value < 0.05 and FC > 1.25).

(G) Feature plots showing the expression of five genes—*CPE*, *EFEMP1*, *GPM6A*, *CDH11*, and *NRG3*—that are highly expressed in cluster 0, as presented in Figure 6E.

(H) *In situ* hybridization for *CD31* together with *GPM6A*, *CDH11*, or *NRG3* in human soleus muscle. *CD31* (Cy5, magenta) and target genes (Cy3, green) are shown. ECs in large blood vessels and capillaries are visualized. Higher magnification views of the regions outlined by black dotted boxes are shown on the right. Red and green arrowheads indicate *CD31* and target gene signals, respectively. Two independent patient samples were analyzed. Scale bars, 50 μm.

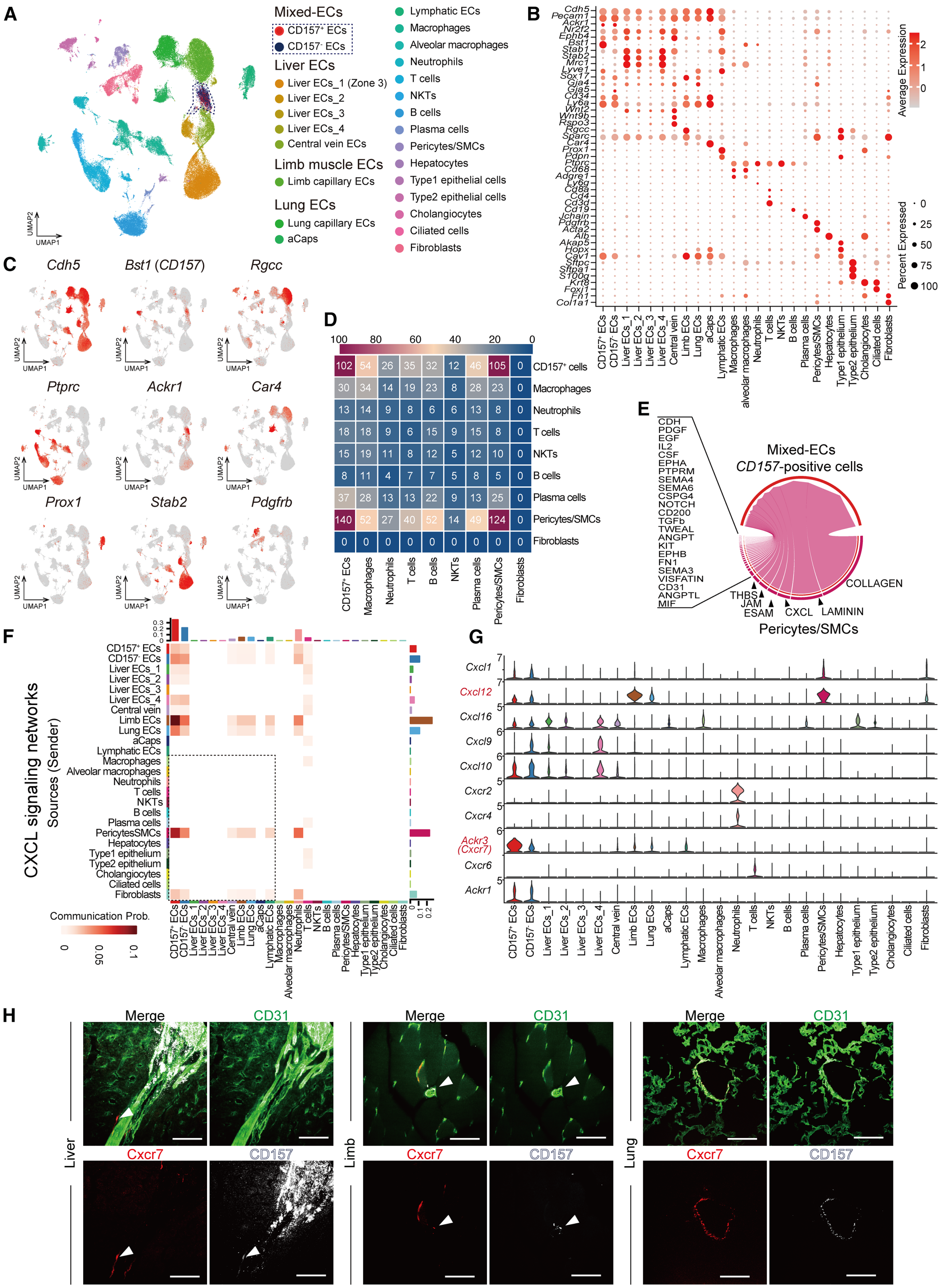

(legend on next page)

CD157-positive ECs also highly expressed *Ackr3* (*Cxcr7*), a gene regulated by *Nfatc1*, whereas pericytes/SMCs expressed *Cxcl12*, the corresponding ligand (Figure 7G). Immunofluorescence staining confirmed CXCR7 expression in CD157$^+$ ECs in mouse liver, hindlimb muscle, and lung tissues (Figure 7H), while *in situ* hybridization demonstrated *CXCL12* expression in perivascular regions, including mural cells (Figures S7A and S7B). Together, these results suggest that CD157$^+$ ECs express CXCR7 and may interact with surrounding cells, including pericytes/SMCs, through CXCL signaling within the microenvironment.

DISCUSSION

An EC population with stem-like features has been proposed previously (Dight et al., 2022), and CD157$^+$ ECs have been shown to display proliferative and vessel-forming capacity (Wakabayashi et al., 2018; 2022). However, scRNA-seq-based characterization of these cells has been limited, and their transcriptomic features have remained unclear.

Here, we used scRNA-seq to analyze multiple mouse tissues and identified a common gene expression profile shared between *CD157*-positive ECs within mixed-EC clusters. These cells expressed *Abcg2* and *Procr* and were enriched for angiogenesis-related pathways, consistent with a stem-like endothelial phenotype. FACS analysis identified CD157$^+$Procr$^+$ ECs as a subset with elevated proliferative capacity, indicating heterogeneity within the CD157$^+$ EC population. Co-expression of CD157 and Procr may mark a proliferative, stem-like EC subset. Mechanistically, these cells were linked to NFAT signaling, as its inhibition reduced proliferation and network formation. NFAT functions downstream of VEGF and is involved in angiogenic regulation, suggesting that it may contribute to the stem-like properties in CD157$^+$ ECs. However, NFAT signaling alone does not sufficiently account for their stem-like characteristics, as NFAT activity broadly regulates endothelial proliferation and function across EC populations.

In humans, we identified a VESC-enriched cluster (hVESC) with high proliferative capacity and a gene expression profile resembling that of mouse *CD157*-positive ECs. Similar to the mouse population, this cluster included cells expressing *CD157*, *PROCR*, and *ABCG2*, although their distribution differed between species. *CD157* expression was more restricted in humans, whereas *PROCR* was more broadly detected but concentrated within the hVESC cluster. *ABCG2* was widely expressed in mice but more confined in humans. *NFATC1* was broadly expressed across ECs, with relatively higher levels in the hVESC cluster. Together, these observations indicate that stem cell-associated features are conserved but not identical across species, and that both interspecies and organ-specific differences require further clarification.

Functionally, Abcg2 acts as a drug efflux transporter. It is expressed in various stem cell populations and underlies the side population phenotype used to enrich tissue stem cells, including VESCs (Goodell et al., 1996; Iba et al., 2019; Naito et al., 2012; 2016). Its drug efflux activity may contribute to the maintenance of a protective stem cell microenvironment. CD157 has been linked to cellular energy metabolism, although its role in stemness remains unclear (Covarrubias et al., 2021), raising the possibility that it influences the metabolic environment supporting stem-like properties. Procr has anticoagulant and cytoprotective functions and has been reported to influence EC cycle progression, suggesting a potential role in regulating proliferative capacity (Chambers et al., 2025; Mohan Rao et al., 2014). The roles of newly identified genes in the hVESC cluster likewise remain to be characterized.

Integration of our EC datasets with publicly available whole-tissue transcriptomic data enabled assessment of potential interactions between EC subclusters and surrounding cell types. This analysis suggested that CD157$^+$ ECs may engage in communication with neighboring cells

Figure 7. *CD157*-positive ECs exhibit specific cell-cell interaction signals in mice
(A) UMAP of our integrated dataset of mouse ECs from three tissues combined with all cell types from a public dataset covering the same three tissues (total: 81,844 cells).
(B) Bubble plots showing the expression of selected marker genes used to define each cluster in Figure 7A.
(C) Feature plots of selected genes projected onto the integrated UMAP shown in Figure 7A.
(D) Heatmap showing the results of cell-cell interaction analysis using CellPhoneDB between the mixed-origin EC cluster and surrounding cell populations from the liver, hindlimb muscle, and lung. The *x* axis represents the sender cell populations, and the *y* axis represents the receiver cell populations. The numbers indicate the total number of detected ligand-receptor interactions.
(E) Circos plot showing ligand-receptor interactions from the pericyte/SMCs cluster to *CD157*-positive ECs within the mixed-EC cluster, as analyzed by CellChat. Interactions are grouped by signaling family.
(F) Heatmap showing communication probabilities between sender-receiver pairs for CXCL signaling networks. The black dotted box indicates signals sent from surrounding cells to EC subpopulations.
(G) Violin plots showing the expression levels of CXCL signaling-related genes across clusters.
(H) Immunofluorescence images of mouse liver, hindlimb muscle, and lung stained for CD31 (green), Cxcr7 (red), and CD157 (white). Scale bars,ss 50 μm.

and indicated a potential role for the CXCL12–CXCR7 signaling axis. We confirmed CXCR7 expression in CD157^{+} ECs by immunostaining. CXCL12–CXCR7 signaling has been reported to be activated by VEGF in tumor ECs and to influence angiogenic properties (Yamada et al., 2015), raising the possibility that VESCs may be primed to initiate angiogenesis in response to stimulation. While this analysis relies primarily on bioinformatic inference and requires further experimental validation, it may provide a useful framework for exploring the functional properties of endothelial subclusters.

More broadly, a limitation of the present study is that our conclusions are largely based on transcriptomic analyses and remain to be validated *in vivo*. Cross-species integration of human and mouse datasets remains methodologically challenging and may introduce potential biases. Thus, findings derived from these integrated datasets, including the cell-cell interaction analyses and the inferred functions of newly identified genes, require cautious interpretation and warrant further experimental confirmation. Our experiments using human CD157^{+} ECs also did not directly establish *in vivo* relevance, nor did they fully address inter-individual variability or potential organ-specific differences. Isolation of CD157^{+} ECs by FACS was also less efficient in human samples than in mice, indicating that technical optimization may be necessary. Nonetheless, we believe that the identification and characterization of human VESCs, including CD157^{+} ECs, represent an important step toward a better understanding of human vascular biology. Moreover, although multiple signaling pathways were identified, their relative importance in mediating interactions between VESCs and neighboring cells and in maintaining the niche remains to be defined. Future studies will be required to clarify how these pathways relate to the stem-like properties and proliferative capacity of VESCs.

In summary, we have demonstrated that CD157-positive ECs exhibit conserved features across multiple tissues. Our scRNA-seq analysis revealed that these cells express high levels of pro-angiogenic factors, even under homeostatic conditions, and that their counterparts are present in human tissues. These findings support the concept of a vascular endothelial stem-like population with potential relevance for vascular regeneration or disruption therapy.

RESOURCE AVAILABILITY

Lead contact

Requests for further information and resources should be directed to and will be fulfilled by the lead contact, Hisamichi Naito (naito.hisamichi@gmail.com).

Materials availability

This study did not generate new unique reagents.

Data and code availability

- The scRNA-seq data are available at the Gene Expression Omnibus (GEO) under accession number GSE303731.
- All original code has been deposited at GitHub and is publicly available at https://github.com/Ivalyne/scRNAseq_CD157VESCs.
- Any additional information required to reanalyze the data reported in this paper is available from the lead contact upon request.

ACKNOWLEDGMENTS

We thank Ms. M. Ishida, Ms. Y. Mitani, Ms. C. Hirose, Ms. T. Yuhi, Ms. M. Oura, and Ms. Kikuta for their technical assistance. We also appreciate the technical support provided by Kanazawa University Environmental Stress Research Center for single-cell RNA library preparation and by Biken Biomics for sequencing. We acknowledge the following financial support for the research presented in this article: the Japan Agency for Medical Research and Development (PRIME JP21gm6210009, FORCE JP24gm4010023, Research Program on Hepatitis JP25fk0210129), the MEXT/JSPS KAKENHI (25K18773, 24K02221, 22H05063, 22H05060, 22K15523, 21K20741, and 20H03435), JST FOREST Program (JPMJFR206A), the Takeda Science Foundation, the Kato Memorial Fund for Incurable Disease Research, and the Kanazawa University SAKIGAKE project.

AUTHOR CONTRIBUTIONS

H.N. conceived the project; T.I., T.W., and H.N. performed the single-cell analysis; T.I., T.W., K.Y., Y.I., and M.H. conducted the cellular and histological experiments; M.S. provided support for the analysis of human samples; A.M., J.M., and N.N. provided additional experimental support; T.I. performed all data analyses; R.H., A.S., and S.F. provided resources; T.I., T.W., and H.N. wrote the manuscript, and H.N. edited the final version; D.M., R.T., and H.A. provided critical input; N.T. and H.N. supervised the research. All authors read and approved the final manuscript.

DECLARATION OF INTERESTS

N.T. serves as a Scientific Advisor for Revascular Bio.

DECLARATION OF GENERATIVE AI AND AI-ASSISTED TECHNOLOGIES IN THE WRITING PROCESS

During the preparation of this work, the authors used ChatGPT to improve language and readability. After using this tool, the manuscript was further edited by a professional English editing service. The authors reviewed and revised the text as needed and take full responsibility for the content of the publication.

STAR★METHODS

Detailed methods are provided in the online version of this paper and include the following:

- KEY RESOURCES TABLE
- EXPERIMENTAL MODEL AND STUDY PARTICIPANT DETAILS

- Mice
- Human samples
- Cell culture
- METHOD DETAILS
  - Mouse cell preparation and cell sorting
  - Human cell preparation and cell sorting
  - Single-cell RNA sequencing
  - Single-cell RNA-seq data analysis
  - Cell culture and primary endothelial colony forming assay
  - Immunostaining
  - *In situ* hybridization (ISH) assay
- QUANTIFICATION AND STATISTICAL ANALYSIS

SUPPLEMENTAL INFORMATION

Supplemental information can be found online at https://doi.org/10.1016/j.stemcr.2026.102931.

Received: September 29, 2025
Revised: April 29, 2026
Accepted: May 4, 2026
Published: June 4, 2026

REFERENCES

Aibar, S., González-Blas, C.B., Moerman, T., Huynh-Thu, V.A., Imrichova, H., Hulselmans, G., Rambow, F., Marine, J.C., Geurts, P., Aerts, J., et al. (2017). SCENIC: single-cell regulatory network inference and clustering. Nat. Methods *14*, 1083–1086.

Barnett, S.N., Cujba, A.M., Yang, L., Maceiras, A.R., Li, S., Kedlian, V.R., Pett, J.P., Polanski, K., Miranda, A.M.A., Xu, C., et al. (2024). An organotypic atlas of human vascular cells. Nat. Med. *30*, 3468–3481.

Binet, F., and Sapieha, P. (2015). ER Stress and Angiogenesis. Cell Metab. *22*, 560–575.

Blanco, R., and Gerhardt, H. (2013). VEGF and Notch in tip and stalk cell selection. Cold Spring Harb. Perspect. Med. *3*, a006569.

Chambers, S.E.J., Guduric-Fuchs, J., Pedrini, E., Bertelli, P.M., Charoensuk, C., Peixoto, E., Pathak, V., Alhamdan, H.I., Xie, R., Krasnodembskaya, A., et al. (2025). Human endothelial colony forming cells (ECFCs) require endothelial protein C receptor (EPCR) for cell cycle progression and angiogenic activity. Angiogenesis *28*, 30.

Chambers, S.E.J., Pathak, V., Pedrini, E., Soret, L., Gendron, N., Guerin, C.L., Stitt, A.W., Smadja, D.M., and Medina, R.J. (2021). Current concepts on endothelial stem cells definition, location, and markers. Stem Cells Transl. Med. *10*, S54–S61.

Covarrubias, A.J., Perrone, R., Grozio, A., and Verdin, E. (2021). NAD(+) metabolism and its roles in cellular processes during ageing. Nat. Rev. Mol. Cell Biol. *22*, 119–141.

Dight, J., Zhao, J., Styke, C., Khosrotehrani, K., and Patel, J. (2022). Resident vascular endothelial progenitor definition and function: the age of reckoning. Angiogenesis *25*, 15–33.

Goodell, M.A., Brose, K., Paradis, G., Conner, A.S., and Mulligan, R.C. (1996). Isolation and functional properties of murine hematopoietic stem cells that are replicating in vivo. J. Exp. Med. *183*, 1797–1806.

Hao, Y., Hao, S., Andersen-Nissen, E., Mauck, W.M., 3rd, Zheng, S., Butler, A., Lee, M.J., Wilk, A.J., Darby, C., Zager, M., et al. (2021). Integrated analysis of multimodal single-cell data. Cell *184*, 3573–3587.e29.

Hie, B., Bryson, B., and Berger, B. (2019). Efficient integration of heterogeneous single-cell transcriptomes using Scanorama. Nat. Biotechnol. *37*, 685–691.

Hsu, Y.C., and Fuchs, E. (2012). A family business: stem cell progeny join the niche to regulate homeostasis. Nat. Rev. Mol. Cell Biol. *13*, 103–114.

Iba, T., Naito, H., Shimizu, S., Rahmawati, F.N., Wakabayashi, T., and Takakura, N. (2019). Isolation of tissue-resident endothelial stem cells and their use in regenerative medicine. Inflamm. Regen. *39*, 9.

Jin, S., Guerrero-Juarez, C.F., Zhang, L., Chang, I., Ramos, R., Kuan, C.H., Myung, P., Plikus, M.V., and Nie, Q. (2021). Inference and analysis of cell-cell communication using CellChat. Nat. Commun. *12*, 1088.

Kalucka, J., de Rooij, L.P.M.H., Goveia, J., Rohlenova, K., Dumas, S.J., Meta, E., Conchinha, N.V., Taverna, F., Teuwen, L.A., Veys, K., et al. (2020). Single-Cell Transcriptome Atlas of Murine Endothelial Cells. Cell *180*, 764–779.e20.

Korsunsky, I., Millard, N., Fan, J., Slowikowski, K., Zhang, F., Wei, K., Baglaenko, Y., Brenner, M., Loh, P.R., and Raychaudhuri, S. (2019). Fast, sensitive and accurate integration of single-cell data with Harmony. Nat. Methods *16*, 1289–1296.

Li, H.Y., Gao, Y.X., Wu, J.C., Li, J.Z., Fu, S.W., and Xu, M.Y. (2024). Single-cell transcriptome reveals a novel mechanism of C-Kit(+)-liver sinusoidal endothelial cells in NASH. Cell Biosci. *14*, 31.

Li, Z., Solomonidis, E.G., Meloni, M., Taylor, R.S., Duffin, R., Dobie, R., Magalhaes, M.S., Henderson, B.E.P., Louwe, P.A., D'Amico, G., et al. (2019). Single-cell transcriptome analyses reveal novel targets modulating cardiac neovascularization by resident endothelial cells following myocardial infarction. Eur. Heart J. *40*, 2507–2520.

Lin, Y., Gil, C.H., Banno, K., Yokoyama, M., Wingo, M., Go, E., Prasain, N., Liu, Y., Hato, T., Naito, H., et al. (2024). ABCG2-Expressing Clonal Repopulating Endothelial Cells Serve to Form and Maintain Blood Vessels. Circulation *150*, 451–465.

Mehrad, B., Keane, M.P., and Strieter, R.M. (2007). Chemokines as mediators of angiogenesis. Thromb. Haemost. *97*, 755–762.

Mohan Rao, L.V., Esmon, C.T., and Pendurthi, U.R. (2014). Endothelial cell protein C receptor: a multiliganded and multifunctional receptor. Blood *124*, 1553–1562.

Naito, H., Iba, T., and Takakura, N. (2020a). Mechanisms of new blood-vessel formation and proliferative heterogeneity of endothelial cells. Int. Immunol. *32*, 295–305.

Naito, H., Kidoya, H., Sakimoto, S., Wakabayashi, T., and Takakura, N. (2012). Identification and characterization of a resident vascular stem/progenitor cell population in preexisting blood vessels. EMBO J. *31*, 842–855.

Naito, H., Wakabayashi, T., Ishida, M., Gil, C.H., Iba, T., Rahmawati, F.N., Shimizu, S., Yoder, M.C., and Takakura, N. (2020b). Isolation of tissue-resident vascular endothelial stem cells from mouse liver. Nat. Protoc. *15*, 1066–1081.

Naito, H., Wakabayashi, T., Kidoya, H., Muramatsu, F., Takara, K., Eino, D., Yamane, K., Iba, T., and Takakura, N. (2016). Endothelial Side Population Cells Contribute to Tumor Angiogenesis and Antiangiogenic Drug Resistance. Cancer Res. *76*, 3200–3210.

Ortolan, E., Augeri, S., Fissolo, G., Musso, I., and Funaro, A. (2019). CD157: From immunoregulatory protein to potential therapeutic target. Immunol. Lett. *205*, 59–64.

Pan, X., Li, X., Dong, L., Liu, T., Zhang, M., Zhang, L., Zhang, X., Huang, L., Shi, W., Sun, H., et al. (2024). Tumour vasculature at single-cell resolution. Nature *632*, 429–436.

Potente, M., Gerhardt, H., and Carmeliet, P. (2011). Basic and therapeutic aspects of angiogenesis. Cell *146*, 873–887.

Profaci, C.P., Harvey, S.S., Bajc, K., Zhang, T.Z., Jeffrey, D.A., Zhang, A.Z., Nemec, K.M., Davtyan, H., O'Brien, C.A., McKinsey, G.L., et al. (2024). Microglia are not necessary for maintenance of blood-brain barrier properties in health, but PLX5622 alters brain endothelial cholesterol metabolism. Neuron *112*, 2910–2921.e7.

Reyfman, P.A., Walter, J.M., Joshi, N., Anekalla, K.R., McQuattie-Pimentel, A.C., Chiu, S., Fernandez, R., Akbarpour, M., Chen, C.I., Ren, Z., et al. (2019). Single-Cell Transcriptomic Analysis of Human Lung Provides Insights into the Pathobiology of Pulmonary Fibrosis. Am. J. Respir. Crit. Care Med. *199*, 1517–1536.

Rodor, J., Chen, S.H., Scanlon, J.P., Monteiro, J.P., Caudrillier, A., Sweta, S., Stewart, K.R., Shmakova, A., Dobie, R., Henderson, B.E.P., et al. (2022). Single-cell RNA sequencing profiling of mouse endothelial cells in response to pulmonary arterial hypertension. Cardiovasc. Res. *118*, 2519–2534.

Schaum, N., Karkanias, J., Neff, N.F., May, A.P., Quake, S.R., Wyss-Coray, T., Darmanis, S., Batson, J., Botvinnik, O., Chen, M.B., et al. (2018). Single-cell transcriptomics of 20 mouse organs creates a Tabula Muris: the tabula muris consortium. Nature *562*, 367–372.

Schindelin, J., Arganda-Carreras, I., Frise, E., Kaynig, V., Longair, M., Pietzsch, T., Preibisch, S., Rueden, C., Saalfeld, S., Schmid, B., et al. (2012). Fiji: an open-source platform for biological-image analysis. Nat. Methods *9*, 676–682.

Seo, S., Fujita, H., Nakano, A., Kang, M., Duarte, A., and Kume, T. (2006). The forkhead transcription factors, Foxc1 and Foxc2, are required for arterial specification and lymphatic sprouting during vascular development. Dev. Biol. *294*, 458–470.

Su, T., Yang, Y., Lai, S., Jeong, J., Jung, Y., McConnell, M., Utsumi, T., and Iwakiri, Y. (2021). Single-Cell Transcriptomics Reveals Zone-Specific Alterations of Liver Sinusoidal Endothelial Cells in Cirrhosis. Cell. Mol. Gastroenterol. Hepatol. *11*, 1139–1161.

Suehiro, J.i., Kanki, Y., Makihara, C., Schadler, K., Miura, M., Manabe, Y., Aburatani, H., Kodama, T., and Minami, T. (2014). Genome-wide approaches reveal functional vascular endothelial growth factor (VEGF)-inducible nuclear factor of activated T cells (NFAT) c1 binding to angiogenesis-related genes in the endothelium. J. Biol. Chem. *289*, 29044–29059.

Trimm, E., and Red-Horse, K. (2023). Vascular endothelial cell development and diversity. Nat. Rev. Cardiol. *20*, 197–210.

Troulé, K., Petryszak, R., Cakir, B., Cranley, J., Harasty, A., Prete, M., Tuong, Z.K., Teichmann, S.A., Garcia-Alonso, L., and Vento-Tormo, R. (2025). CellPhoneDB v5: inferring cell-cell communication from single-cell multiomics data. Nat. Protoc. *20*, 3412–3440.

Wakabayashi, T., and Naito, H. (2023). Cellular heterogeneity and stem cells of vascular endothelial cells in blood vessel formation and homeostasis: Insights from single-cell RNA sequencing. Front. Cell Dev. Biol. *11*, 1146399.

Wakabayashi, T., Naito, H., Iba, T., Nishida, K., and Takakura, N. (2022). Identification of CD157-Positive Vascular Endothelial Stem Cells in Mouse Retinal and Choroidal Vessels: Fluorescence-Activated Cell Sorting Analysis. Investig. Ophthalmol. Vis. Sci. *63*, 5.

Wakabayashi, T., Naito, H., Suehiro, J.I., Lin, Y., Kawaji, H., Iba, T., Kouno, T., Ishikawa-Kato, S., Furuno, M., Takara, K., et al. (2018). CD157 Marks Tissue-Resident Endothelial Stem Cells with Homeostatic and Regenerative Properties. Cell Stem Cell *22*, 384–397.e6.

Wickham, H., Averick, M., Bryan, J., Chang, W., McGowan, L., François, R., Grolemund, G., Hayes, A., Henry, L., Hester, J., et al. (2019). Welcome to the tidyverse. J. Open Source Softw. *4*, 1686.

Wu, T., Hu, E., Xu, S., Chen, M., Guo, P., Dai, Z., Feng, T., Zhou, L., Tang, W., Zhan, L., et al. (2021). clusterProfiler 4.0: A universal enrichment tool for interpreting omics data. Innovation *2*, 100141.

Xiong, X., Kuang, H., Ansari, S., Liu, T., Gong, J., Wang, S., Zhao, X.Y., Ji, Y., Li, C., Guo, L., et al. (2019). Landscape of Intercellular Crosstalk in Healthy and NASH Liver Revealed by Single-Cell Secretome Gene Analysis. Mol. Cell *75*, 644–660.e5.

Yamada, K., Maishi, N., Akiyama, K., Towfik Alam, M., Ohga, N., Kawamoto, T., Shindoh, M., Takahashi, N., Kamiyama, T., Hida, Y., et al. (2015). CXCL12-CXCR7 axis is important for tumor endothelial cell angiogenic property. Int. J. Cancer *137*, 2825–2836.

Yilmaz, Ö.H., Katajisto, P., Lamming, D.W., Gültekin, Y., Bauer-Rowe, K.E., Sengupta, S., Birsoy, K., Dursun, A., Yilmaz, V.O., Selig, M., et al. (2012). mTORC1 in the Paneth cell niche couples intestinal stem-cell function to calorie intake. Nature *486*, 490–495.

Yu, Q.C., Song, W., Wang, D., and Zeng, Y.A. (2016). Identification of blood vascular endothelial stem cells by the expression of protein C receptor. Cell Res. *26*, 1079–1098.

Zheng, G.X.Y., Terry, J.M., Belgrader, P., Ryvkin, P., Bent, Z.W., Wilson, R., Ziraldo, S.B., Wheeler, T.D., McDermott, G.P., Zhu, J., et al. (2017). Massively parallel digital transcriptional profiling of single cells. Nat. Commun. *8*, 14049.

STAR★METHODS

KEY RESOURCES TABLE

REAGENT or RESOURCE	SOURCE	IDENTIFIER
**Antibodies**		
Purified Anti-Mouse CD16/CD32 (2.4G2)	CYTEC Biosciences	Cat# 70-0161-U100; RRID: AB_2621487
Brilliant Violet 421 anti-mouse CD31	BioLegend	Cat# 102423; RRID: AB_2562186
CD45 Monoclonal Antibody (30-F11), FITC	Thermo Fisher Scientific	Cat# 11-0451-82; RRID: AB_465050
APC anti-mouse CD157 (BST-1)	BioLegend	Cat# 140208; RRID: AB_10901172
PE anti-mouse CD201 (EPCR)	BioLegend	Cat# 141504; RRID: AB_10895909
Human TruStain FcX (Fc Receptor Blocking Solution)	BioLegend	Cat# 422302; RRID: AB_2818986
APC anti-human CD31	BioLegend	Cat# 303116; RRID: AB_1877152
Brilliant Violet 421 anti-human CD45	BioLegend	Cat# 304032; RRID: AB_2561357
BST-1/CD157, Human (FITC conj.)	MBL	Cat# D036-4; RRID: AB_590906
Purified Rat Anti-Mouse CD31	BD Biosciences	Cat# 553370; RRID: AB_394816
Anti-Alpha Smooth Muscle Actin	BioLegend	Cat# 904601; RRID:AB_2565041
PE anti-human/mouse CXCR7	BioLegend	Cat# 331104; RRID: AB_1227619
CD31 (PECAM-1) (F8M3S) Rabbit Monoclonal Antibody	Cell Signaling Technology	Cat# 95462; RRID: AB_3739873
Alexa Fluor 647-AffiniPure Fab Fragment Goat Anti-Rabbit IgG	Jackson ImmunoResearch Labs	Cat# 111-607-008; RRID: AB_2632470
Goat anti-Mouse IgG2a Cross-Adsorbed Secondary Antibody, Alexa Fluor 568	Thermo Fisher Scientific	Cat# A-21134; RRID: AB_2535773
Goat anti-Mouse IgG1 Cross-Adsorbed Secondary Antibody, Alexa Fluor 488	Thermo Fisher Scientific	Cat# A-21121; RRID: AB_2535764
Goat anti-Rat IgG (H + L) Cross-Adsorbed Secondary Antibody, Alexa Fluor 488	Thermo Fisher Scientific	Cat# A-11006; RRID: AB_2534074
Goat anti-Rat IgG (H + L) Secondary Antibody, Biotin	Thermo Fisher Scientific	Cat# 31830; RRID: AB_228355
Goat Anti-Mouse IgG Antibody (H + L), Biotinylated	Vector Laboratories	Cat# AI-9200-1.5; RRID: AB_3107016
**Biological samples**		
Human soleus muscle	Juntendo University Hospital	N/A
**Chemicals, peptides, and recombinant proteins**		
Cyclosporine (Sandimmun®)	Novartis Pharma	NDC 00078-0109
DAPI	Thermo Fisher Scientific	Cat# D1306
Propidium Iodide Solution	BioLegend	Cat# 421301
Dispase II	Thermo Fisher Scientific	Cat# 17105041

(*Continued on next page*)

Continued

REAGENT or RESOURCE	SOURCE	IDENTIFIER
Collagenase	Wako	Cat# 034-22363
Collagenase Type II	Worthington Biochemical	Cat# LS004176
Collagenase type XI	Sigma-Aldrich	Cat# H3506
Soybean trypsin inhibitor	Worthington Biochemical	Cat# LS003571
Lyophilized elastase	Worthington Biochemical	Cat# LS002292
Hyaluronidase type I	Sigma-Aldrich	Cat# H4034
ACK Lysing Buffer	Lonza	Cat# 10-548E
Percoll	Cytiva	Cat# 17089102
Debris Removal Solution	Miltenyi Biotec	Cat# 130-109-398
Bovine serum albumin	Sigma-Aldrich	Cat# A9418-10G
Minimum Essential Medium Eagle-alpha modification	Sigma-Aldrich	Cat# M8042-500ML
Fetal bovine serum	Nichirei Biosciences	Cat# 175012
L-glutamine	Thermo Fisher Scientific	Cat# 25030081
Penicillin/streptomycin	Thermo Fisher Scientific	Cat# 15140122
RPMI-1640	Sigma-Aldrich	Cat# R8758
VEGF 165, Human, Recombinant	PeproTech	Cat# 100-20-10ug
2-mercaptoethanol	Thermo Fisher Scientific	Cat# 21985023
EGM-2 Endothelial Cell Growth Medium-2 BulletKit	Lonza	Cat# CC-3162
Opal 480 Reagent Pack	Akoya Biosciences	Cat# FP1500001KT
Opal 570 Reagent Pack	Akoya Biosciences	Cat# FP1488001KT
Opal 620 Reagent Pack	Akoya Biosciences	Cat# FP1495001KT
New Hematoxylin Type G	Muto Pure Chemicals	Cat# 30161
VECTASTAIN Elite ABC Kit	Vector Laboratories	Cat# PK-6100
optimal cutting temperature (OCT) compound	Sakura Finetek	Cat# 45833
**Critical commercial assays**		
RNAscope 2.5 Duplex Reagent Kit	Advanced Cell Diagnostics	Cat# 322430
Chromium Next GEM Single Cell 3′ Kit v3.1	10x Genomics	Cat# PN-1000269
**Deposited data**		
Mouse and Human Single-Cell RNA seq data	This study	GEO: GSE303731
Mouse Liver Single-Cell RNA seq data	Su et al. (2021)	GEO: GSE147581
Mouse Liver Single-Cell RNA seq data	Li et al. (2024)	GEO: GSE225786
Mouse Liver Single-Cell RNA seq data	Xiong et al. (2019)	GEO: GSE129516
Mouse Brain Single-Cell RNA seq data	Profaci et al. (2024)	GEO: GSE267591
Mouse Heart Single-Cell RNA seq data	Li et al. (2019)	GEO: GSE132880
Mouse Lung Single-Cell RNA seq data	Rodor et al. (2022)	GEO: GSE154959

(*Continued on next page*)

Continued

REAGENT or RESOURCE	SOURCE	IDENTIFIER
Mouse Lung Single-Cell RNA seq data	Reyfman et al. (2019)	GEO: GSE121611
Mouse Single-Cell RNA seq data	Schaum et al. (2018)	GEO: GSE109774
Human Vascular Atlas Single-Cell RNA seq data	Barnett et al. (2024)	https://www.vascularcellatlas.org/
**Experimental models: Cell lines**		
Mouse: OP9	Riken BRC	RCB1124
**Experimental models: Organisms/strains**		
C57BL/6	Japan SLC	N/A
**Software and algorithms**		
ImageJ/Fiji v2.16	Schindelin et al. (2012)	https://imagej.net/
10x Genomics Cell Ranger v7.1.0	Zheng et al. (2017)	https://www.10xgenomics.com/
Seurat v4.4.0	Hao et al. (2021)	https://satijalab.org/seurat/
tidyverse v2.0	Wickham et al. (2019)	https://tidyverse.org/
clusterProfiler v4.7.1.002	Wu et al. (2021)	https://bioconductor.org/packages/clusterProfiler/
Scanorama v1.7.4	Hie et al. (2019)	https://github.com/brianhie/scanorama
SCENIC v1.3.1	Aibar et al. (2017)	https://github.com/aertslab/SCENIC
harmony v1.2.0	Korsunsky et al. (2019)	https://CRAN.R-project.org/package=harmony
CellPhoneDB v5.0.1	Troulé et al. (2025)	https://github.com/ventolab/CellphoneDB
CellChat v1.6.1	Jin et al. (2021)	https://github.com/jinworks/CellChat
FlowJo software	BD Biosciences	https://www.flowjo.com/
Photoshop CC	Adobe Systems	https://www.adobe.com/
R v4.2.2	R Core Team, 2022	https://www.r-project.org/
reticulate v1.44.1	GitHub	https://rstudio.github.io/reticulate/
org.Hs.e.g.,.db v3.16	Bioconductor	https://bioconductor.org/packages/org.Hs.eg.db/
org.Mm.e.g.,.db v3.16	Bioconductor	https://bioconductor.org/packages/org.Mm.eg.db/
Orthology.e.g.,.db v3.16	Bioconductor	https://bioconductor.org/packages/Orthology.eg.db/
gplots v3.1.3.1	CRAN	https://CRAN.R-project.org/package=gplots
pheatmap v1.0.12	CRAN	https://CRAN.R-project.org/package=pheatmap
EnhancedVolcano v1.16	GitHub	https://github.com/kevinblighe/EnhancedVolcano
loomR v0.2.1.9000	GitHub	https://github.com/mojaveazure/loomR
SCopeLoomR v0.13	GitHub	https://github.com/aertslab/SCopeLoomR
AngioTool 2.0	GitHub	https://github.com/jbendtsen/AngioTool-Batch
ktplots v2.4.0	GitHub	https://github.com/zktuong/ktplots
**Other**		
RNAscope™ Probe- Hs-GPM6A	Advanced Cell Diagnostics	Cat# 470601
RNAscope™ Probe- Hs-CDH11	Advanced Cell Diagnostics	Cat# 448381

(*Continued on next page*)

Continued

REAGENT or RESOURCE	SOURCE	IDENTIFIER
RNAscope™ Probe- Hs-NRG3	Advanced Cell Diagnostics	Cat# 893971-C1
RNAscope™ Probe- Hs-PECAM1-01-C2	Advanced Cell Diagnostics	Cat# 487381-C2
RNAscope™ Probe-Mm-Bst1-01-C1	Advanced Cell Diagnostics	Cat# 1868681-C1
RNAscope™ Probe-Mm-Pecam1-C2	Advanced Cell Diagnostics	Cat# 316721-C2
RNAscope™ Probe-Mm-Cxcl12-C4	Advanced Cell Diagnostics	Cat# 422711-C4

EXPERIMENTAL MODEL AND STUDY PARTICIPANT DETAILS

Mice

All animal experiments were approved by the Animal Care and Use Committees of Osaka University and Kanazawa University and were performed in accordance with the Science Council of Japan Guidelines for Proper Conduct of Animal Experiments. C57BL/6J mice were obtained from the Japan SLC. Animals were maintained in a clean conventional condition at Kanazawa University on a 12-hour light/dark cycle.

Human samples

Human skeletal muscle specimens were collected from patients at Juntendo University in accordance with protocols approved by the Research Ethics Committee of Juntendo University School of Medicine (approval no. H16-0101) and the Medical Ethics Committee of Kanazawa University (approval no. 2022-001 [82639]). Written informed consent was obtained from all participants. All samples were de-identified prior to downstream analyses. The specimens were used for flow cytometry, endothelial cell isolation, scRNA sequencing, cell culture assays, immunofluorescence analysis, and *in situ* hybridization assays.

Cell culture

OP9 cells (RIKEN Cell Bank, Tsukuba, Japan) were maintained in Minimum Essential Medium Eagle-alpha modification (αMEM) (Sigma-Aldrich, Tokyo, Japan) supplemented with 20% fetal bovine serum (FBS) (Nichirei Biosciences, Tokyo, Japan), 2 mM l-glutamine (Thermo Fisher Scientific, Waltham, MA, USA), and 1% penicillin/streptomycin (p/s) (Life Technologies, Tokyo, Japan).

METHOD DETAILS

Mouse cell preparation and cell sorting

C57BL/6J mice (8 to 10 weeks old) were purchased from Japan SLC (Hamamatsu, Japan). Cells were isolated as previously described (Naito et al., 2020b). Briefly, organs were excised, minced, and digested with dispase II (Thermo Fisher Scientific), collagenase (Wako, Osaka, Japan), and type II collagenase (Worthington Biochemical Corp., Lakewood, NJ, USA) at 37°C with shaking. The cell suspension was filtered through 40 μm strainers, and erythrocytes were lysed using ACK Lysing Buffer (Lonza Japan, Tokyo, Japan). Cells were then treated with Fc blocker (clone 2.4G2, CYTEC Biosciences, Fremont, CA, USA), followed by surface antigen staining with anti-CD31 (clone 390, BioLegend, San Diego, CA, USA), anti-CD45 (clone 30-F11, Thermo Fisher Scientific), anti-CD157 (clone BP-3, BioLegend), and/or anti-CD201 (clone RCR-16, BioLegend) antibodies. Propidium iodide (PI, BioLegend) was added prior to FACS analysis to enable exclusion of dead cells. The stained cells were analyzed and sorted using a FACSAria SORP (BD Biosciences, San Jose, CA, USA) or CytoFLEX SRT (Beckman Coulter, Brea, CA, USA). Data were analyzed using FlowJo software (BD Biosciences).

Human cell preparation and cell sorting

Soleus muscle tissue was obtained from patients undergoing lower limb amputation and the cut edge was used for analysis. Immediately after resection, the samples were washed with saline and stored at 4°C in phosphate-buffered saline (PBS) containing 4% FBS (Nichirei Biosciences). For *in situ* hybridization, tissue was immediately fixed in 10% formalin. For single-cell analysis, the tissue was minced into small pieces, digested with dispase II, and then further digested in an enzyme cocktail containing 3 mg/mL collagenase type II, 0.15 mg/mL collagenase type XI (Sigma-Aldrich), 0.25 mg/mL

soybean trypsin inhibitor (Worthington Biochemical Corp.), 0.1875 mg/mL lyophilized elastase (Worthington Biochemical Corp.), and 0.24 mg/mL hyaluronidase type I (Sigma-Aldrich) in 4% FBS/PBS supplemented with 1 mM $CaCl_2$. The cell suspension was filtered through 40 μm strainers, and erythrocytes were lysed using ACK Lysing Buffer. Cells were then treated with Fc blocker (BioLegend), followed by surface antigen staining with anti-CD31 (clone WM59, BioLegend) and anti-CD45 (clone HI30, BioLegend). The stained cells were gated on FSC/SSC to exclude debris, and doublets and PI-positive dead cells were excluded. $CD31^{+}CD45^{-}$ endothelial cells (ECs) were then sorted using a CytoFLEX SRT. For cell culture assays, the tissue was minced into small pieces and digested with dispase II, collagenase and type II collagenase at 37°C with shaking. The resulting cell suspension was filtered through 40 μm strainers, and erythrocytes were lysed using ACK Lysing Buffer. Remaining debris was removed using Debris Removal Solution (Miltenyi Biotec, Bergisch Gladbach, Germany) and the cells were further enriched by density gradient centrifugation using Percoll (Cytiva, Tokyo, Japan). The cells were then treated with Fc blocker, followed by surface antigen staining with anti-CD31 and anti-CD45 and anti-CD157 (clone RF3, MBL, Tokyo, Japan). The stained cells were gated on FSC/SSC to exclude debris, and doublets and PI-positive dead cells were excluded. $CD31^{+}CD45^{-}CD157^{+}$ and $CD31^{+}CD45^{-}CD157^{-}$ ECs were then sorted using a CytoFLEX SRT.

Single-cell RNA sequencing

Single-cell capture and library preparation were performed using the Chromium Next GEM Single Cell 3′ Kit v3.1 with Dual Index (10x Genomics, Pleasanton, CA, USA), following the manufacturer's instructions. Fresh mouse-derived and patient-derived single-cell suspensions were diluted to 1000 cells/μL in PBS containing 0.1% bovine serum albumin (BSA) (Sigma-Aldrich). The sequencing library was constructed according to the manufacturer's instructions. Sequencing was performed on a DNBSEQ-G400 platform (MGI Tech, Shenzhen, China) by Biken Biomics Co., Ltd. (Osaka, Japan), using 28 bp × 100 bp paired-end reads with dual indexing. Sequenced reads were demultiplexed and mapped to the GRCm39 (mouse) or GRCh38 (human) reference genome (Ensembl v98) using CellRanger v7.1.0 (10x Genomics).

Single-cell RNA-seq data analysis

Single-cell RNA-seq data were processed and analyzed using the Seurat package (version 4.4.0). The percentage of mitochondrial unique molecular identifier (UMI) counts was calculated using the Seurat function *PercentageFeatureSet*. Cells with feature counts or mitochondrial gene UMI count ratios exceeding either twice the peak value or the 95th percentile in their respective histograms were excluded as part of quality control filtering. Gene expression matrices were normalized using the *LogNormalize* function with default parameters. Cell cycle regression was performed using the *CellCycleScoring* function, which assigns S and G2/M scores and classifies cells into G1, S, or G2/M phases.

For data integration and batch correction, two complementary workflows were employed, and their results were compared for consistency and robustness. In the first workflow, data were integrated using an optimized Seurat standard RPCA integration pipeline, followed by batch correction using the Harmony algorithm (Korsunsky et al., 2019). Briefly, highly variable genes (top 2000) were identified using *FindVariableFeatures*, and integration features were selected across datasets using *SelectIntegrationFeatures* with default parameters. Unwanted variation—including mitochondrial UMI counts, RNA feature counts, total RNA counts, and cell cycle scores—was regressed out using *ScaleData*. Principal component analysis (PCA) was then performed using *RunPCA*. Integration anchors were identified using *FindIntegrationAnchors* with *reduction = "rpca"*, and datasets were integrated using these anchors. After integration, *ScaleData* was again used to regress out unwanted variation, and highly variable genes (top 3000) were re-identified using *FindVariableFeatures*. PCA was repeated with *RunPCA*, and the optimal number of dimensions was determined using a modified ElbowPlot approach (Harvard Chan Bioinformatics Core). Batch correction was performed using the Harmony algorithm via *RunHarmony*. Dimensionality reduction and clustering were performed using *RunUMAP*, *FindNeighbors*, and *FindClusters*. In the second workflow, data were normalized and technical and biological variation was regressed using *SCTransform*. Integration features were selected using *SelectIntegrationFeatures*, and the datasets were subsequently integrated using the *Scanorama* algorithm (version 1.7.4).

For the integration of mouse and human single-cell RNA-seq data from muscle tissue, genes commonly expressed in both datasets were extracted. The mouse gene expression matrix was then converted to human gene symbols prior to integration. Specifically, genome-wide annotation packages for both mice and humans, along with an orthology mapping package, were used to convert mouse gene identifiers to their human orthologs. After conversion, the gene expression matrices from the two species were compared. Only genes detected in both datasets were retained for further analysis; all others were excluded.

Gene ontology (GO) pathway enrichment analysis was performed using the *ClusterProfiler* package (version 4.7.1.002). Adjusted p-values were used to determine significance. Cluster-specific transcription factor activity was analyzed using the *SCENIC* package (version 1.3.1). Cell–cell interaction analyses were conducted using *CellPhoneDB* (version 5.0.1) and *CellChat* (version 1.6.1), and visualization of *CellPhone*DB results was performed using the *ktplots* package (version 2.4.0).

Cell culture and primary endothelial colony forming assay

Primary mouse ECs were isolated and plated on the OP9 stromal cells in 24-well plates at a density of 3×10^3 cells/well for CD157 and CD201 EC culture experiments. The cells were cultured in RPMI-1640 (Sigma-Aldrich) supplemented with 10% FBS, 10 ng/mL vascular endothelial growth factor (VEGF) (PeproTech, Rocky Hill, NJ, USA), 0.1% 2-mercaptoethanol (Thermo Fisher Scientific), and 1% p/s. Primary human ECs were isolated and seeded onto OP9 stromal cells in 96-well plates at a density of 5×10^3 cells/well. Both $CD157^+$ and $CD157^-$ EC fractions were cultured under identical conditions in complete EGM-2 medium supplemented with 10% FBS and 10 ng/mL VEGF. The cells were fixed for immunostaining after 10 days of culture.

Immunostaining

For confocal microscopy images, sections were prepared as previously reported (Wakabayashi et al., 2018). In brief, mouse liver, lung, and hind limb tissues, as well as human soleus muscle tissue, were collected, fixed in 4% paraformaldehyde/PBS (PFA/PBS), washed with PBS, embedded in optimal cutting temperature (OCT) compound (Sakura Finetek, Tokyo, Japan), sectioned (10 or 40 μm), and mounted on MAS-coated slide glass (#S9115, Matsunami Glass, Osaka, Japan). The mouse sections were stained with anti-CD31 monoclonal antibody (clone MEC13.3, BD Biosciences), anti-CD157 monoclonal antibody (clone BP-3, BioLegend), anti-CD201 monoclonal antibody (clone RCR-16, BioLegend), anti-αSMA monoclonal antibody (clone 1A4, BioLegend) and/or anti-Cxcr7 monoclonal antibody (clone 8F11-M16, BioLegend). Alexa Fluor 488-conjugated anti-rat IgG (Thermo Fisher Scientific), Alexa Fluor 594-conjugated anti-rat IgG (Thermo Fisher Scientific), and/or Alexa Fluor 568-conjugated anti-mouse IgG2a (Thermo Fisher Scientific) were used as secondary antibodies. The human sections were stained with anti-CD31 monoclonal antibody (clone F8M3S, Cell Signaling Technology, Danvers, MA, USA), anti-CD157 monoclonal antibody (clone RF3, MBL), and anti-αSMA monoclonal antibody (clone 1A4, BioLegend). Alexa Fluor 647-conjugated anti-rabbit IgG (Jackson ImmunoResearch Laboratories, West Grove, PA, USA), Alexa Fluor 568-conjugated anti-mouse IgG2a (Thermo Fisher Scientific) and Alexa Fluor 488-conjugated anti-rat IgG1 (Thermo Fisher Scientific) were used as secondary antibodies. The sections were visualized using the Andor Dragonfly 200 system (Oxford Instruments, High Wycombe, UK) equipped with Fusion Stitcher software and built on a Nikon ECLIPSE Ti2 inverted microscope (Nikon Corporation, Tokyo, Japan). Images were processed using Adobe Photoshop CC software (Adobe Systems, San Jose, CA, USA). All images shown are representative of more than three independent experiments. For immunohistochemistry of colony-formation assays, anti-CD31 antibody was used for staining and biotin-conjugated polyclonal anti-rat immunoglobulin (IgG) (Thermo Fisher Scientific) was used as the secondary antibody for mouse samples, whereas biotin-conjugated anti-mouse IgG antibody (Vector Laboratories, Newark, CA, USA) was used for human samples. Biotinylated secondary antibodies were developed using Elite-ABC kits (Vector Laboratories). Samples were visualized using an IX83 microscope equipped with an automated stage (Olympus Life Sciences, Tokyo, Japan). The vascular area was quantified using ImageJ, and the number of branches and average network lengths were calculated using AngioTool 2.0.

In situ hybridization (ISH) assay

For human tissue, *in situ* hybridization (ISH) was performed on formalin-fixed, paraffin-embedded (FFPE) sections using the RNAscope 2.5 Duplex Reagent Kit (Advanced Cell Diagnostics, Newark, CA, USA) according to the manufacturer's protocol. Briefly, for human tissues, soleus muscle was collected from the cut edge of resected tissue from patients undergoing lower limb amputation. Samples were fixed in 10% neutral buffered formalin, dehydrated in ethanol, and embedded in paraffin. Sections (4 μm thick) were processed using standard pretreatment procedures. Probes specific for GPM6A mRNA (RNAscope™ Probe-Hs-GPM6A), CDH11 mRNA (RNAscope™ Probe-Hs-CDH11), NRG3 mRNA (RNAscope™ Probe-Hs-NRG3), and PECAM1 mRNA (RNAscope™ Probe-Hs-PECAM1-O1-C2) were obtained from the manufacturer and hybridized using the HybEZ™ Hybridization System (Advanced Cell Diagnostics). Signals were detected using green horseradish peroxidase (HRP)-based and red alkaline phosphatase (AP)-based chromogenic assays. Tissues were counterstained with New Hematoxylin Type G (Muto Pure Chemicals, Tokyo, Japan), and bright-field images were captured using a IX83 microscope.

For mouse tissues, multiplex fluorescence ISH was performed on liver, hindlimb muscle, and lung sections using the RNAscope Multiplex Fluorescent Reagent Kit v2 (Advanced Cell Diagnostics) with probes targeting Pecam1 (RNAscope™ Probe-Mm-Pecam1-C2), Bst1 (RNAscope™ Probe-Mm-Bst1-O1-C1), and Cxcl12 (RNAscope™ Probe-Mm-Cxcl12-C4). Fluorescently labeled sections were imaged by confocal microscopy using a Dragonfly 200 system.

QUANTIFICATION AND STATISTICAL ANALYSIS

All experiments were performed with at least three biological replicates unless otherwise noted. All data are presented as means ± SEM. Statistical comparisons between two groups were conducted using the Steel–Dwass–Critchlow–Fligner test in the open-source package R (v4.2.2). P values of $P < 0.05$ (*$P < 0.05$; **$P < 0.005$; ***$P < 0.0005$) were considered to indicate statistical significance. Replicates are indicated in the figure legends. All graphs were generated using R.

Stem Cell Reports, Volume *21*

Supplemental Information

CD157$^+$ vascular endothelial stem cells represent a conserved sub-population with an angiogenic gene expression profile

Tomohiro Iba, Taku Wakabayashi, Rie Ito, Ai Sugawara, Satoshi Fujimura, Mika Sawane, Kazuaki Yoshioka, Aya Matsui, Jun-ichi Morishige, Naoto Nagata, Yukinobu Ito, Masafumi Horie, Daichi Maeda, Rica Tanaka, Hitoshi Ando, Nobuyuki Takakura, and Hisamichi Naito

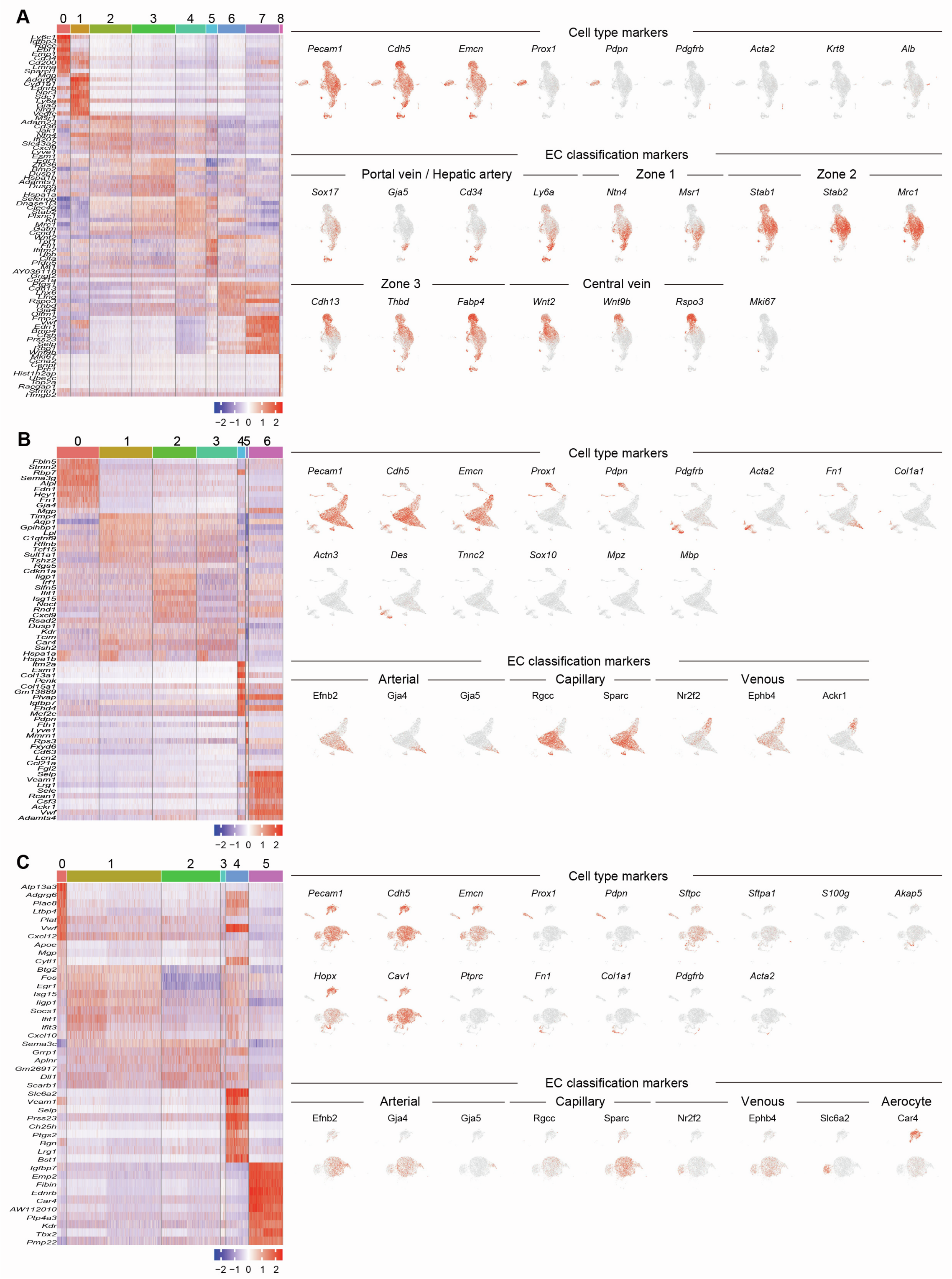

Figure S1. Characterization of EC subclusters in each mouse tissue

Heatmaps (left panels) showing the top 10 differentially expressed genes in each cluster of CD31$^+$CD45$^-$ sorted cells from mouse liver (**A**), hindlimb muscle (**B**), and lung (**C**), as shown in **Figure 1**. Feature plots of selected cell type annotation markers and endothelial cell (EC) subpopulation markers are shown on the right.

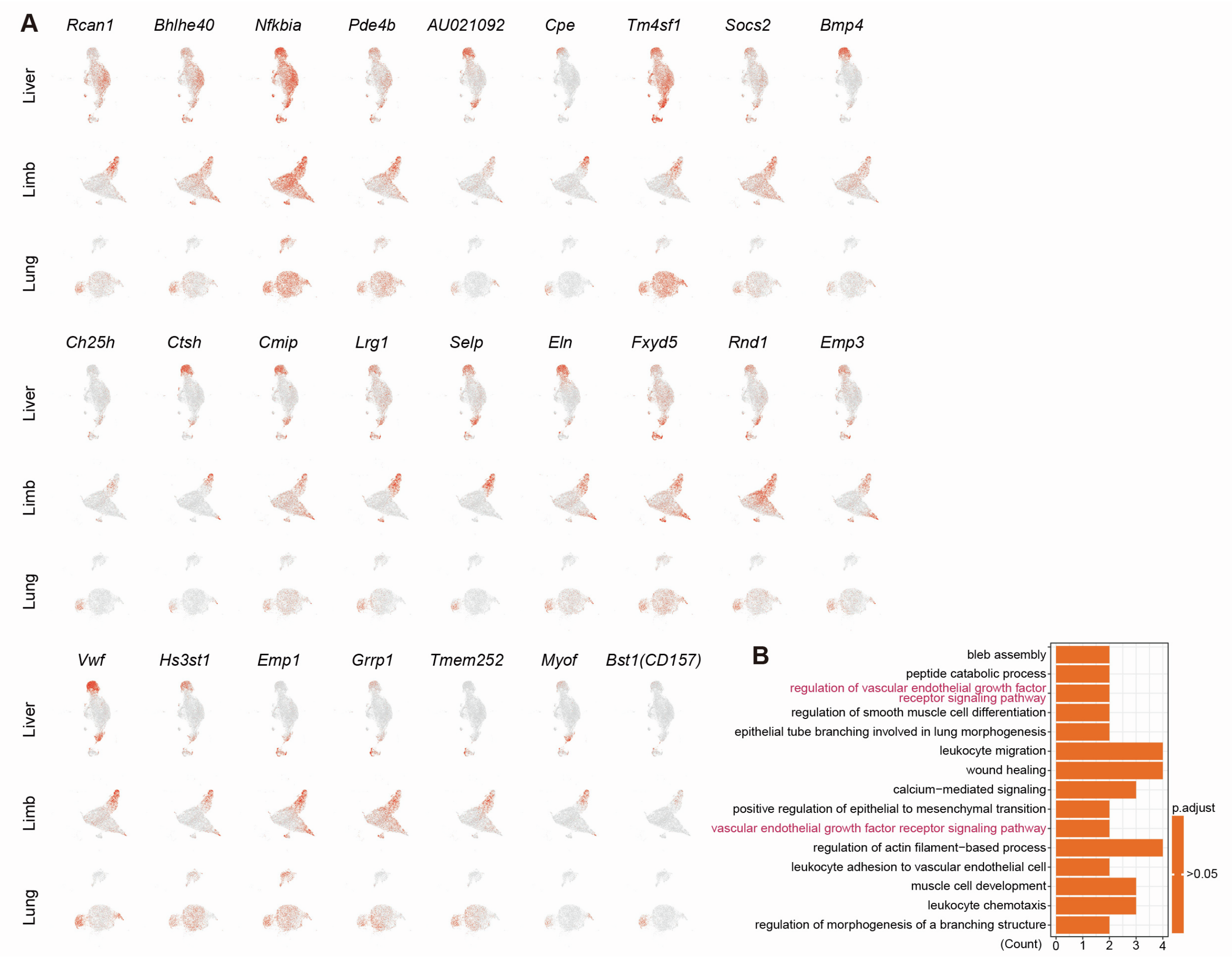

Figure S2. Analysis of genes shared by *CD157*-positive ECs across mouse tissues

A. Feature plots showing the expression of genes commonly expressed in *CD157*-positive ECs across mouse tissues (identified in **Figure 2C**) in the mouse liver, hindlimb muscle, and lung.

B. GO analysis of genes shown in **Figure S2A**.

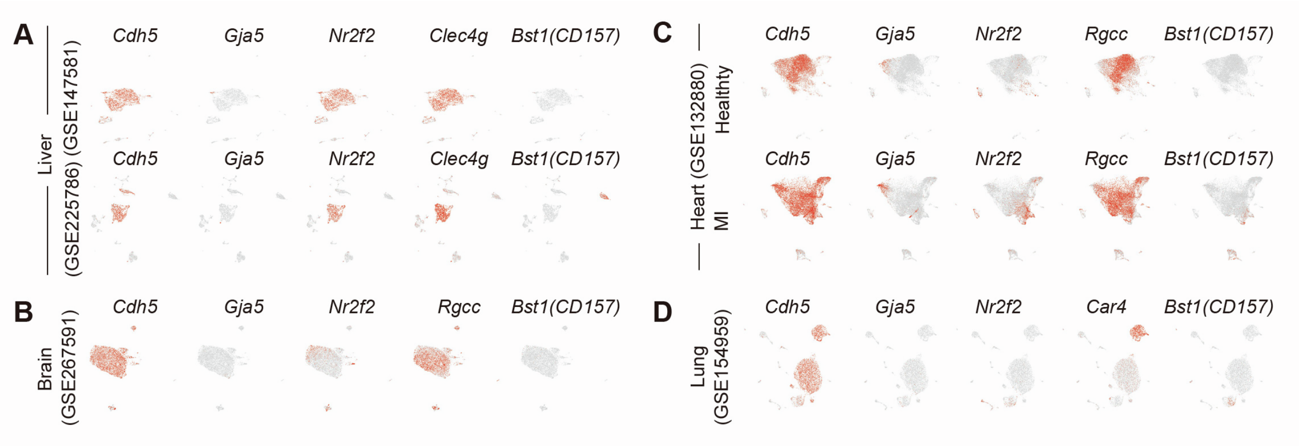

Figure S3. Reanalysis of public mouse scRNA-seq datasets

Feature plots showing the expression of the pan-endothelial marker *Cdh5*; arterial marker *Gja5*; venous marker *Nr2f2*; organ-specific EC markers—*Clec4g* for liver sinusoidal ECs, *Rgcc* for heart capillary ECs, and *Car4* for lung aCaps—as well as *Bst1* (*CD157*), in the liver (**A**), brain (**B**) heart under healthy and myocardial infarction (MI) conditions (**C**), and lung (**D**). This analysis was based on publicly accessible mouse scRNA-seq datasets obtained from the following repositories: liver (GSE147581, GSE225786), brain (GSE267591), heart (GSE132880), and lung (GSE154959).

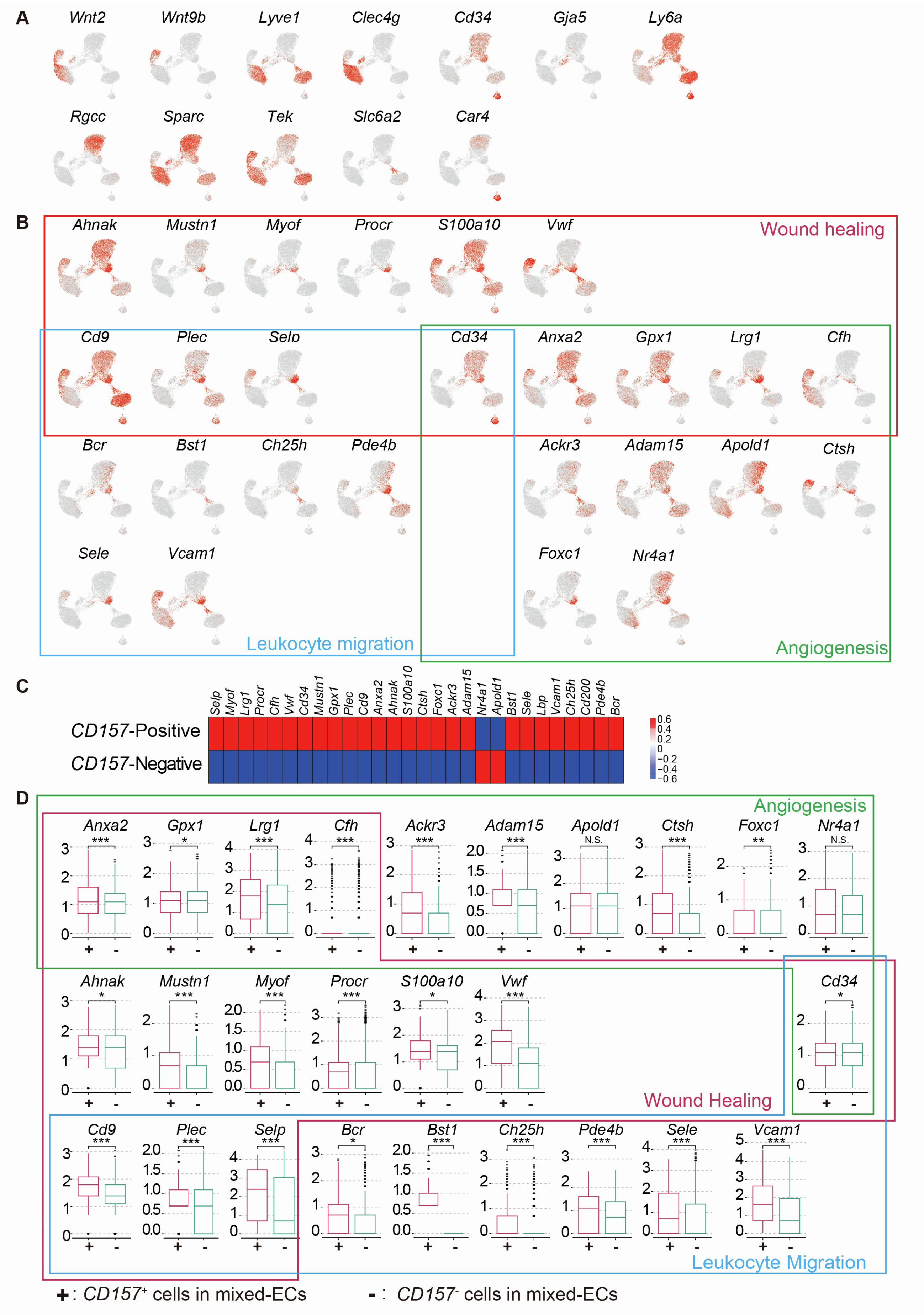
A
Wnt2
Wnt9b
Lyve1
Clec4g
Cd34
Gja5
Ly6a
Rgcc
Sparc
Tek
Slc6a2
Car4
B
Wound healing
Ahnak
Mustn1
Myof
Procr
S100a10
Vwf
Cd9
Plec
Selp
Cd34
Anxa2
Gpx1
Lrg1
Cfh
Bcr
Bst1
Ch25h
Pde4b
Ackr3
Adam15
Apold1
Ctsh
Sele
Vcam1
Foxc1
Nr4a1
Leukocyte migration
Angiogenesis
C
Selp Myof Lrg1 Procr Cfh Vwf Cd34 Mustn1 Gpx1 Plec Cd9 Anxa2 Ahnak S100a10 Ctsh Foxc1 Ackr3 Adam15 Nr4a1 Apold1 Bst1 Sele Lbp Vcam1 Ch25h Cd200 Pde4b Bcr
CD157-Positive
CD157-Negative
0.6
0.4
0.2
0
−0.2
−0.4
−0.6
D
Angiogenesis
Anxa2 ***
Gpx1 *
Lrg1 ***
Cfh ***
Ackr3 ***
Adam15 ***
Apold1 N.S.
Ctsh ***
Foxc1 **
Nr4a1 N.S.
Ahnak *
Mustn1 ***
Myof ***
Procr ***
S100a10 *
Vwf ***
Cd34 *
Wound Healing
Cd9 ***
Plec ***
Selp ***
Bcr *
Bst1 ***
Ch25h ***
Pde4b ***
Sele ***
Vcam1 ***
Leukocyte Migration
+ -
+: CD157+ cells in mixed-ECs
-: CD157- cells in mixed-ECs

Figure S4. Characterization of *CD157*-positive and *CD157*-negative ECs using integrated EC datasets

A. Feature plots showing the expression of central vein markers (*Wnt2* and *Wnt9b*), sinusoidal EC markers (*Lyve1* and *Clec4g*), portal vein EC marker *Cd34*, arterial EC marker *Gja5*, capillary markers (*Rgcc* and *Sparc*), pulmonary general capillary (gCap) marker (*Tek*), pulmonary venous marker (*Slc6a2*), and aerocyte marker (*Car4*) are projected onto the Uniform Manifold Approximation and Projection (UMAP) plots of ECs integrated from three mouse tissues.

B. Feature plots showing the expression of genes associated with the *CD157*-positive cell-specific pathway identified in **Figure 3H**. Genes related to wound healing are outlined in red, those related to leukocyte migration in blue, and those related to angiogenesis in green.

C. Heatmap of genes shown in **Figure 3G**, comparing *CD157*-positive and *CD157*-negative ECs within the mixed-EC cluster.

D. Box plots showing expression of genes detected by pathway analysis in **Figure 3H**, based on the gene set defined in **Figure 3G**, comparing *CD157*-positive and *CD157*-negative ECs within the mixed-EC cluster. Genes related to wound healing are outlined in red, those related to leukocyte migration in blue, and those related to angiogenesis in green. *** $p < 0.0005$, ** $p < 0.005$, *$p < 0.05$.

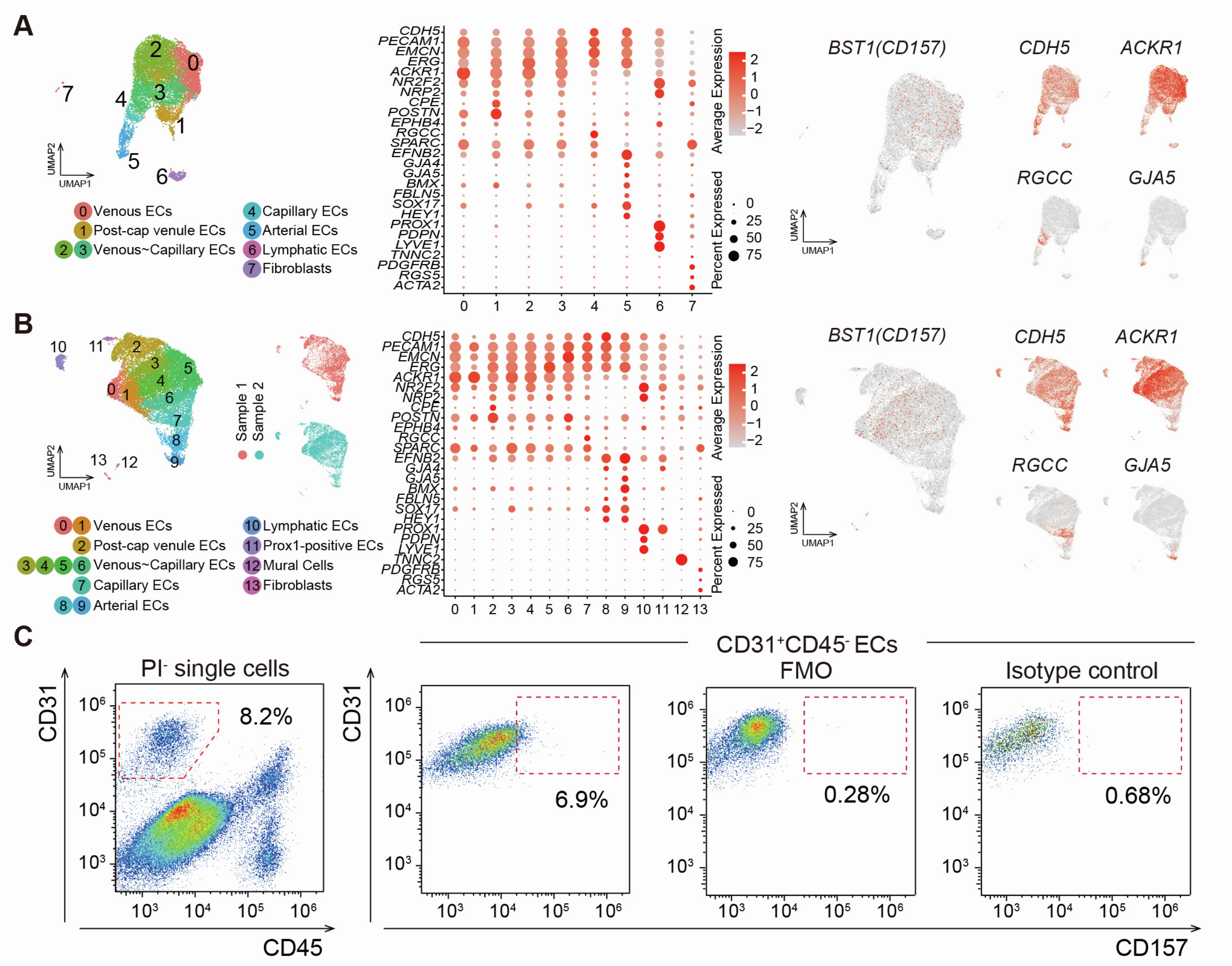

Figure S5. Bioinformatic characterization of human muscle EC single-cell data and FACS profiling of CD157⁺ ECs

A. scRNA-seq analysis of an independent dataset of human muscle CD31⁺CD45⁻ cells, separate from that used in **Figure 5**. (Left) UMAP plots and clustering of 12,915 cells. (Middle) Bubble plots showing expression of canonical marker genes across clusters. (Right) Feature plots of *BST1* (*CD157*), *CDH5*, *ACKR1*, *RGCC*, and *GJA5*.

B. Integrated scRNA-seq analysis of a human muscle CD31⁺CD45⁻ cell dataset shown in **Figure 5** and the independent dataset shown in **Figure S5A**. (Left) UMAP plots and clustering of the integrated 17,873 cells. (Middle) Bubble plots showing expression of canonical marker genes across clusters. (Right) Feature plots of *BST1* (*CD157*), *CDH5*, *ACKR1*, *RGCC*, and *GJA5*.

C. Representative flow cytometry dot plots of cells isolated from human lower limb muscle, showing CD157 antibody staining with fluorescence minus one (FMO) and isotype controls. The red gate indicates CD157⁺ cells used for cell culture assays.

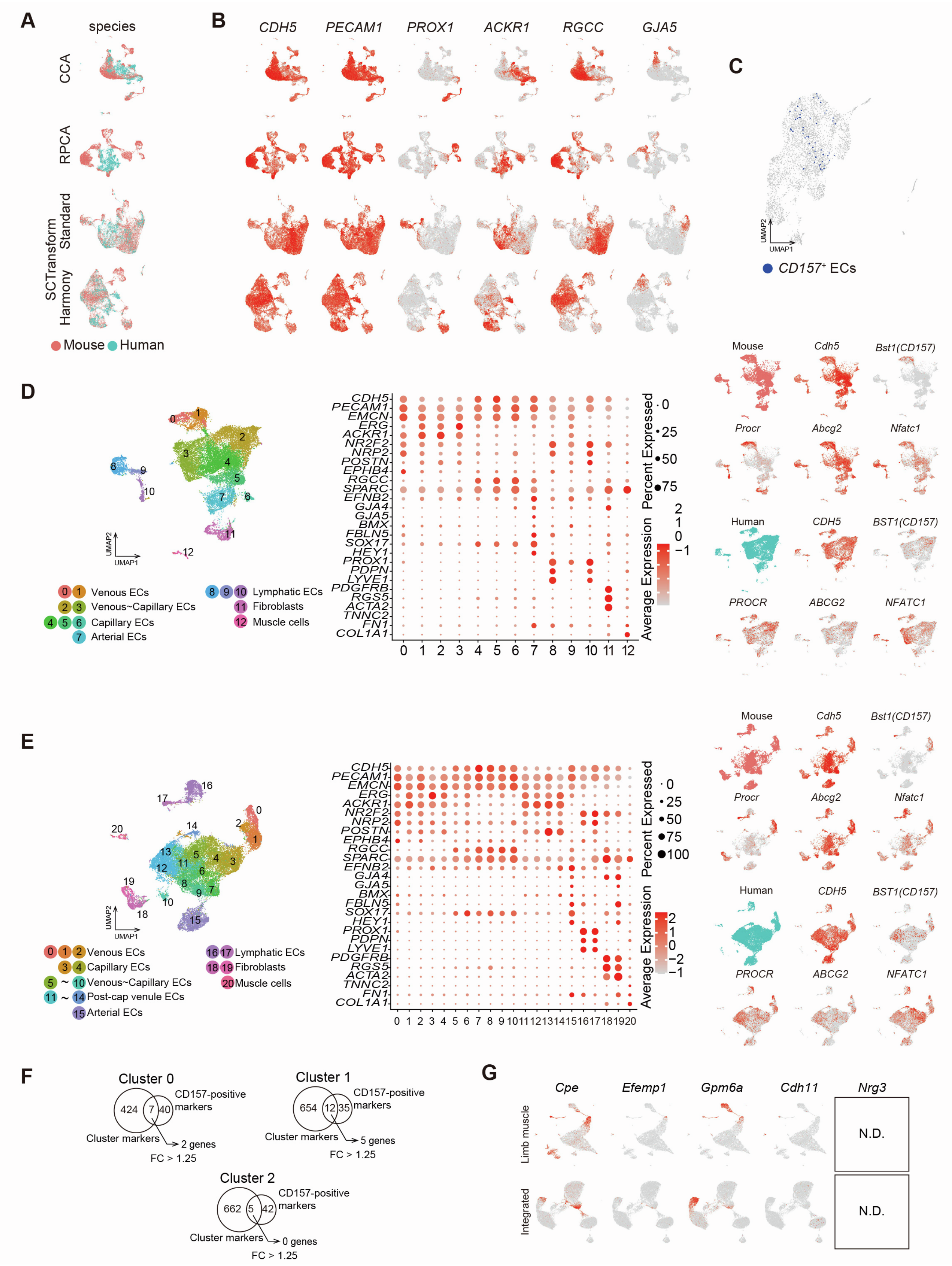

A
species
CCA
RPCA
SCTransform Standard
SCTransform Harmony
Mouse
Human
B
CDH5
PECAM1
PROX1
ACKR1
RGCC
GJA5
C
UMAP2
UMAP1
CD157+ ECs
D
0 1 Venous ECs
2 3 Venous~Capillary ECs
4 5 6 Capillary ECs
7 Arterial ECs
8 9 10 Lymphatic ECs
11 Fibroblasts
12 Muscle cells
Percent Expressed
Average Expression
Mouse
Cdh5
Bst1(CD157)
Procr
Abcg2
Nfatc1
Human
CDH5
BST1(CD157)
PROCR
ABCG2
NFATC1
E
0 1 2 Venous ECs
3 4 Capillary ECs
5 ~ 10 Venous~Capillary ECs
11 ~ 14 Post-cap venule ECs
15 Arterial ECs
16 17 Lymphatic ECs
18 19 Fibroblasts
20 Muscle cells
F
Cluster 0
424
7
40
CD157-positive markers
Cluster markers
2 genes
FC > 1.25
Cluster 1
654
12
35
CD157-positive markers
Cluster markers
5 genes
FC > 1.25
Cluster 2
662
5
42
CD157-positive markers
Cluster markers
0 genes
FC > 1.25
G
Cpe
Efemp1
Gpm6a
Cdh11
Nrg3
Limb muscle
Integrated
N.D.
N.D.

Figure S6. Comparison of human–mouse integration methods and gene expression similarity

A. UMAP plots of human and mouse datasets integrated using multiple methods. Dot colors indicate species origin.

B. Feature plots of the indicated genes used to define EC subsets, projected onto the UMAP plots shown in **Figure S6A.**

C. Feature plots of *CD157*-positive cells extracted from clusters 0, 1, and 2 of the integrated dataset and re-projected onto the human UMAP shown in **Figure 5A**.

D. Integrated scRNA-seq analysis combining a human muscle CD31^{+}CD45^{-} cell dataset used in **Figure S5A** (12,915 cells; distinct from the dataset used in **Figure 6**) with the mouse hindlimb CD31^{+}CD45^{-} cell dataset used in **Figure 6** (12,681 cells). (Left) UMAP plots and clustering of the integrated data. (Middle) Bubble plots showing expression of canonical marker genes across clusters.(Right) Feature plots of indicated genes projected onto species-split UMAPs of the integrated dataset.

E. Integrated scRNA-seq analysis combining the two human datasets used in **Figure S5B** (total 17,873 cells; from **Figures 5** and **S5A**) with the mouse hindlimb CD31^{+}CD45^{-} cell dataset used in **Figure 6** (12,681 cells). (Left) UMAP plots and clustering of the integrated data.(Middle) Bubble plots showing expression of canonical marker genes across clusters. (Right) Feature plots of indicated genes projected onto species-split UMAPs of the integrated dataset.

F. Venn diagrams showing the overlap of upregulated genes between each cluster and *CD157*-positive ECs.

G. Feature plots of Cluster 0 signature genes in the mouse limb muscle dataset (top) and in the mouse integrated dataset (bottom).

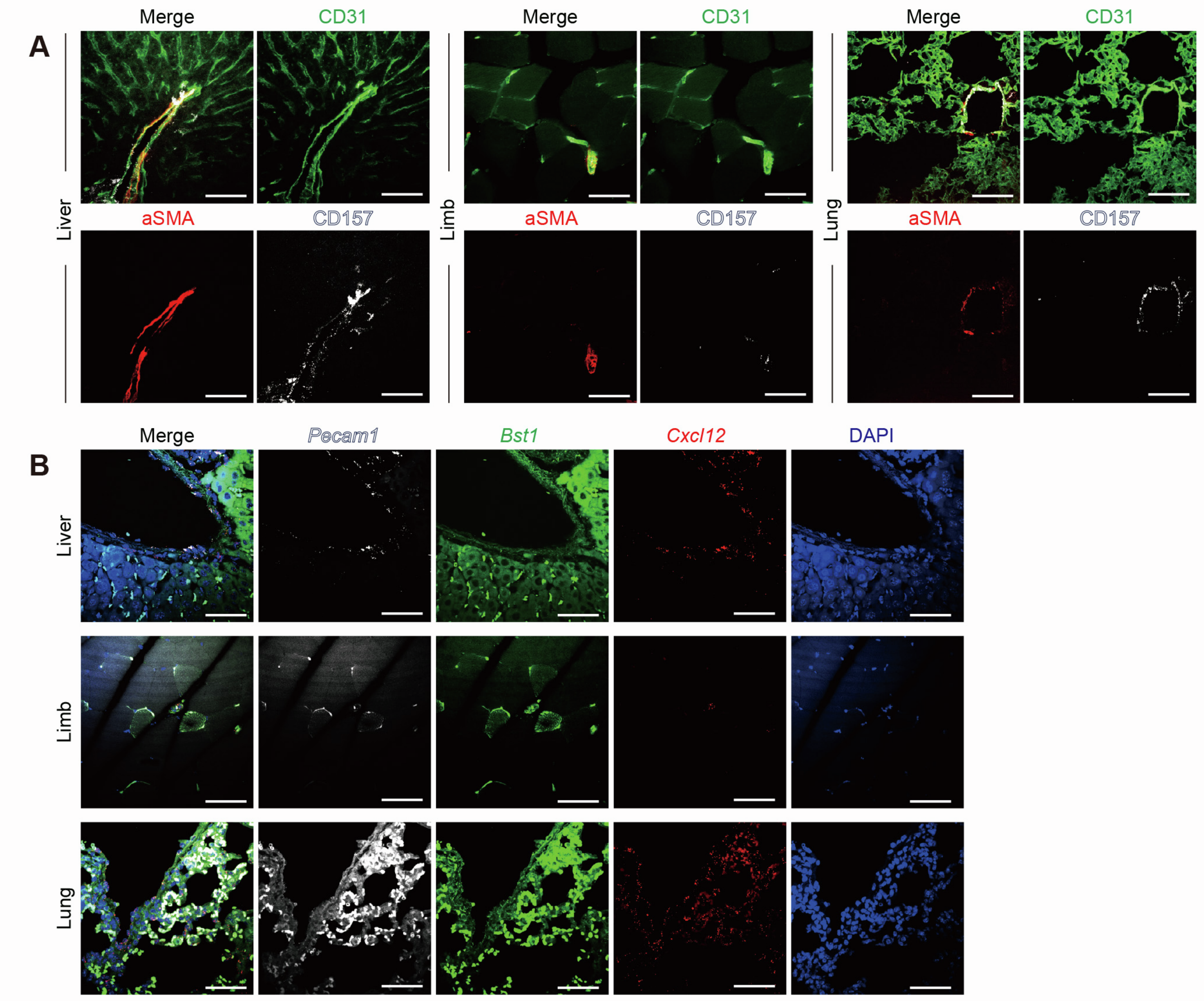

Figure S7. Perivascular expression of CXCL12 by in situ hybridization

A. Immunofluorescence images of mouse liver, hindlimb muscle, and lung tissues stained for CD31 (green), αSMA (red), and CD157 (white), showing smooth muscle cells surrounding the CD31⁺ endothelium, with CD157 expression observed in a subset of ECs. Scale bars: 50 μm.

B. *In situ* hybridization for *Pecam1* together with *Bst1* and *Cxcl12* in mouse liver, hindlimb muscle, and lung tissues. *Cxcl12* signals are observed in regions corresponding to the αSMA-positive areas shown in **Figure S7A**. Signals for *Pecam1* (white), *Bst1* (green), *Cxcl12* (red), and DAPI (blue) are shown. Scale bars: 50 μm.

Cluster 0	Cluster 1	Cluster 2
CPE	SELE	DOCK11
EFEMP1	CSF3	PLCXD3
MGP	IL6	HLA-DQA1
GPM6A	VCAM1	NET1
CDH11	SLC4A7	LHX6
CRTAC1	TMEM70	KRT7
MMRN1	ADAMTS3	PDE7B
TMOD1	CYP1B1	C7
PTGIS	CFAP161	
PLA2G4A	RGS16	
NRG3	PVT1	
CARMIL1	TRIB1	
MEIS2	STC1	
TIMP1	ENKUR	
RAB11FIP1	C7	
C7	SLC16A7	
SLCO2A1	SELP	
PTGS2	SLC6A16	
CYP1B1	RHOU	
ACKR3	LRG1	
ST8SIA6	LUCAT1	
SRPX	RHBDL2	
HS3ST2	KDM6B	
HMGN3	HAPLN3	
ITGA10		
NR2F2-AS1		
CTSC		
PLCXD3		
PON2		
LDLRAD3		
IL13RA1		
BMX		
NPAS2		
RYR3		
COL8A1		
CAMK1D		
HRCT1		
CSGALNACT1		
PDLIM3		
C11orf96		
SGMS2		

Table S1. Differentially expressed genes in human EC clusters 0, 1, and 2

List of differentially expressed genes (DEGs) in human EC clusters 0, 1, and 2 from the integrated dataset. DEGs were selected based on the criteria of fold change (FC) > 1.25 and expression detected in less than 50% of cells in the comparison group (pct.2 < 0.5).

	CD157+ ECs	CD157- ECs	Liver ECs_1	Liver ECs_2	Liver ECs_3	Liver ECs_4	Central vein	Limb capillary ECs	Lung capillary ECs	aCaps	Lymphatic ECs	Macrophages	alveolar macrophages	Neutrophils	T cells	NKTs	B cells	Plasma cells	Pericytes/SMCs	Hepatocytes	Type1 epithelium	Type2 epithelium	Cholangiocytes	Ciliated cells	Fibroblasts
CD157+ ECs	0.02293434	0.01670262	0	0	0	0	0.0011429	0.002642633	0.002624418	0	0.002595711	0	0	0.01812082	0.00215042	0	0	0	0	0	0	0	0	0	0
CD157- ECs	0.03936366	0.03410016	0	0	0	0	0.00130212	0.003010162	0.00298942	0	0.002956733	0	0	0.02541389	0.00270447	0	0	0	0	0	0	0	0	0	0
Liver ECs_1	0.00899635	0.01024146	0	0	0	0	0	0	0	0	0	0	0	0	0.01121818	0	0	0	0	0	0	0	0	0	0
Liver ECs_2	0.00355573	0.00404838	0	0	0	0	0	0	0	0	0	0	0	0	0.0039095	0	0	0	0	0	0	0	0	0	0
Liver ECs_3	0	0	0	0	0	0	0	0	0	0	0	0	0	0	0	0	0	0	0	0	0	0	0	0	0
Liver ECs_4	0.01992636	0.02266427	0	0	0	0	0	0	0	0	0	0	0	0	0.00433206	0	0	0	0	0	0	0	0	0	0
Central vein	0.00322002	0.00366634	0	0	0	0	0	0	0	0	0	0	0	0	0.00715847	0	0	0	0	0	0	0	0	0	0
Limb capillary ECs	0.10122095	0.0494083	0	0	0	0	0.00895295	0.02049096	0.020352226	0	0.020133521	0	0	0.0422096	0	0	0	0	0	0	0	0	0	0	0
Lung capillary ECs	0.04530587	0.02024101	0	0	0	0	0.00397006	0.009145687	0.009083054	0	0.008984337	0	0	0.01509177	0	0	0	0	0	0	0	0	0	0	0
aCaps	0	0	0	0	0	0	0	0	0	0	0	0	0	0	0.0024613	0	0	0	0	0	0	0	0	0	0
Lymphatic ECs	0	0	0	0	0	0	0	0	0	0	0	0	0	0	0	0	0	0	0	0	0	0	0	0	0
Macrophages	0	0	0	0	0	0	0	0	0	0	0	0	0	0	0.00430749	0	0	0	0	0	0	0	0	0	0
alveolar macrophages	0	0	0	0	0	0	0	0	0	0	0	0	0	0	0	0	0	0	0	0	0	0	0	0	0
Neutrophils	0	0	0	0	0	0	0	0	0	0	0	0	0	0	0	0	0	0	0	0	0	0	0	0	0
T cells	0	0	0	0	0	0	0	0	0	0	0	0	0	0	0	0	0	0	0	0	0	0	0	0	0
NKTs	0	0	0	0	0	0	0	0	0	0	0	0	0	0	0	0	0	0	0	0	0	0	0	0	0
B cells	0	0	0	0	0	0	0	0	0	0	0	0	0	0	0	0	0	0	0	0	0	0	0	0	0
Plasma cells	0	0	0	0	0	0	0	0	0	0	0	0	0	0	0.00191311	0	0	0	0	0	0	0	0	0	0
Pericytes/SMCs	0.08351968	0.04150379	0	0	0	0	0.00709958	0.016288335	0.016177584	0	0.016003007	0	0	0.05100127	0	0	0	0	0	0	0	0	0	0	0
Hepatocytes	0	0	0	0	0	0	0	0	0	0	0	0	0	0	0	0	0	0	0	0	0	0	0	0	0
Type1 epithelium	0	0	0	0	0	0	0	0	0	0	0	0	0	0	0.00445953	0	0	0	0	0	0	0	0	0	0
Type2 epithelium	0	0	0	0	0	0	0	0	0	0	0	0	0	0	0.00201727	0	0	0	0	0	0	0	0	0	0
Cholangiocytes	0	0	0	0	0	0	0	0	0	0	0	0	0	0	0	0	0	0	0	0	0	0	0	0	0
Ciliated cells	0	0	0	0	0	0	0	0	0	0	0	0	0	0	0	0	0	0	0	0	0	0	0	0	0
Fibroblasts	0.02113242	0.01257721	0	0	0	0	0.00140049	0.003237147	0.003214846	0	0.003179702	0	0	0.02793045	0	0	0	0	0	0	0	0	0	0	0

Table S2. Communication probability scores of CXCL signaling between clusters

A table of communication probability scores. Scores between *CD157*-positive ECs and pericytes/SMCs, as well as those between *CD157*-negative ECs and pericytes/SMCs, are highlighted.
